# Epigenome-wide analysis in West Africans identifies DNA methylation markers for circulating adiponectin

**DOI:** 10.1016/j.ebiom.2026.106192

**Published:** 2026-03-06

**Authors:** Muhulo Muhau Mungamba, Johanna Wijburg, Eva L. van der Linden, Felix P. Chilunga, Ayo P. Doumatey, Amy R. Bentley, Charles F. Hayfron-Benjamin, Constance R. Sewani-Rusike, Benedicta N. Nkeh-Chungag, Rexford S. Ahima, Charles Agyemang, Peter Henneman, Adebowale A. Adeyemo, Charles N. Rotimi, Karlijn A.C. Meeks

**Affiliations:** a Department of Public and Occupational Health, Amsterdam University Medical Centers, University of Amsterdam, Amsterdam Public Health Research Institute, Amsterdam, the Netherlands; b Department of Human Biology, Faculty of Medicine and Health Sciences, Walter Sisulu University, Mthatha, South Africa; c Department of Vascular Medicine, Amsterdam University Medical Centers, University of Amsterdam, Amsterdam Cardiovascular Sciences, Amsterdam, the Netherlands; d Center for Research on Genomics and Global Health, National Human Genome Research Institute, National Institutes of Health, Bethesda, MD, USA; e Departments of Physiology and Anaesthesia/Critical Care, University of Ghana Medical School, Korle Bu Teaching Hospital, Ghana; f Department of Biological and Environmental Sciences, Faculty of Natural Sciences, Walter Sisulu University, Mthatha, South Africa; g Division of Endocrinology, Diabetes and Metabolism, Department of Medicine, The Johns Hopkins University School of Medicine, Baltimore, MD, USA; h Department of Human Genetics, Epigenetics, Amsterdam Reproduction and Development, Research Institute, Amsterdam University Medical Centers, Amsterdam, the Netherlands; i Division of Endocrinology, Diabetes and Nutrition, Department of Medicine, University of Maryland School of Medicine, Baltimore, MD, USA

**Keywords:** DNA methylation, Epigenome-wide association study, Adiponectin, Adipokine, Sub-Saharan Africa

## Abstract

**Background:**

Adiponectin is a circulating adipokine involved in energy metabolism and inflammation, with reported protective effects against cardiometabolic diseases such as type 2 diabetes (T2D) and early kidney disease. However, its regulation remains poorly understood. This study aimed to identify epigenetic loci associated with adiponectin levels.

**Methods:**

DNA methylation was profiled using Illumina 450K and EPIC (850K) arrays in 315 Ghanaians (RODAM-Pros study) and 593 Nigerians (AADM study). Differentially methylated positions (DMPs) were identified using linear regression models adjusted for age, sex, BMI, blood cell proportions, and technical covariates. Analyses were stratified by T2D status and cohort, then meta-analysed to identify DMPs associated with adiponectin across T2D status (combining participants with-and-without diabetes). RNA-seq data on 77 blood, 49 subcutaneous adipose tissue (SAT), and 55 skeletal muscle samples from the AADM study were used to identify eQTMs for identified DMPs.

**Findings:**

We identified three epigenome-wide significant DMPs: cg03546163 (Z-score = 5.76, p ≤ 0.001, 5′UTR of *FKBP5*), cg02561343 (Z-score = 5.11, p ≤ 0.001, within *UST*), and cg23969380 (Z-score = 5.13, p ≤ 0.001, *ADGRD1* body). cg03546163 was an eQTM for *PLA2G12B* in SAT (beta = −0.039, FDR = 0.047), cg02561343 for *PSMD8* (beta = −11.85, FDR = 0.029) and *TECR* (beta = −9.48, FDR = 0.029) in SAT, and cg23969380 for *HIGD2AP1* (beta = −0.095, FDR = 0.024) in blood. These genes have been reported to be involved in lipid metabolism (*PLA2G12B* and *TECR*), proteasomal degradation (*PSMD8*), and cellular stress-responses (*HIGD2AP1*).

**Interpretation:**

This epigenome-wide study of adiponectin in sub-Saharan African populations identified DNA methylation loci potentially involved in adiponectin regulation through lipid-metabolism, inflammation, proteostasis, and stress–response pathways. These findings provide a foundation for replication and further investigation to improve understanding of the role of adiponectin in cardiometabolic-health.

**Funding:**

10.13039/100000002National Institutes of Health and 10.13039/501100000781European Research Council.

**Research in context**

**Evidence before this study**

We searched PubMed for studies published between January 1, 2000, and June 30, 2025, using the MeSH terms “Adiponectin,” “DNA methylation,” and “Epigenetics” to assess the extent of current knowledge on the epigenetic regulation of circulating adiponectin. We also queried the EWAS Atlas, a curated knowledgebase of epigenome-wide association studies (EWAS), for published adiponectin EWAS. Our search identified a substantial number of candidate gene studies, particularly examining methylation at the ADIPOQ promoter region in the context of maternal and foetal programming, obesity, and type 2 diabetes. However, only one EWAS, conducted among European and African Americans in the United States, performed a genome-wide investigation of DNA methylation in relation to adiponectin levels. This EWAS reported a suggestive association at a single CpG site in *CPT1A*, a locus commonly implicated in obesity and metabolic disease, potentially reflecting downstream effects or confounding. To date, no EWAS have reported statistically robust associations that elucidate biological mechanisms underlying adiponectin regulation. Overall, existing evidence is limited in scope, population diversity, and genomic coverage, leaving key knowledge gaps regarding the epigenetic architecture of adiponectin.

**Added value of this study**

By analysing DNA methylation in two well-characterised African cohorts (Nigerians and Ghanaians), we identified three CpG sites significantly associated with adiponectin levels at the genome-wide level. We also detected population-specific epigenome-wide CpG sites, which may reflect differences in environmental exposures or genetic background. Integration with RNA sequencing data from adipose tissue, blood, and skeletal muscle indicated that the three primary CpG sites function as expression quantitative trait methylation (eQTM) sites for genes involved in lipid metabolism, proteasome activity, and cellular stress responses. These findings extend understanding of adiponectin regulation beyond candidate regions and provide mechanistic insights into its role in metabolic health.

**Implications of all the available evidence**

Our study provides epigenetic insights into the regulation of adiponectin, highlighting broader molecular networks involving lipid metabolism, inflammation, and cellular stress responses. In addition to enhancing understanding of disease mechanisms, these results underscore the importance of ancestry- and context-specific epigenetic studies in uncovering biological pathways relevant to cardiometabolic health and potential targets for prevention or therapeutic intervention. Further studies in additional populations are needed to distinguish shared from ancestry-specific signals and to explore their causal roles and clinical relevance.

## Introduction

Adiponectin is an adipokine primarily secreted by adipose tissue that plays a central role in regulating glucose homoeostasis, lipid metabolism, insulin sensitivity, and inflammation. Its anti-inflammatory and anti-atherosclerotic properties, including the suppression of foam cell formation, contribute to cardiometabolic protection.^1^^,^^2^ In epidemiological studies, adiponectin levels have been inversely associated with the risk of type 2 diabetes (T2D),^3^ cardiovascular diseases and related disorders.^4^ However, the regulation of adiponectin levels remains poorly understood, particularly in populations underrepresented in biomedical research, including genomics.

Sub-Saharan African populations face a high and rising burden of T2D and cardiometabolic diseases.^5^, ^6^, ^7^ These populations exhibit unique genetic and environmental factors that influence cardiometabolic traits but remain underrepresented in epigenetic studies. Although heritability estimates for adiponectin levels range from 30% to 70%, and genome-wide association studies (GWAS) have identified several associated loci, these findings account for only a fraction of the variability in circulating adiponectin levels.^8^, ^9^, ^10^, ^11^ Environmental factors such as smoking,^12^ alcohol use,^13^^,^^14^ physical activity,^15^ and pollution^16^ also influence adiponectin levels through mechanisms that are not well understood. Investigating the epigenetic regulation of adiponectin in sub-Saharan Africans is of particular importance as these populations are experiencing rapid lifestyle changes due to urbanisation, including shifts in diet, physical activity, and exposure to environmental stressors^17^ that can influence adiponectin regulation and its effects on cardiometabolic health.

Recent evidence suggests that epigenetic mechanisms, particularly DNA methylation, play a significant role in regulating adiponectin expression. DNA methylation, which involves the addition of methyl groups to cytosine residues within CpG dinucleotides, often results in gene suppression.^18^ For example, hypermethylation of adiponectin (*ADIPOQ*) promoter region has been reported to be linked to reduced adiponectin expression^19^ and associated cardiometabolic traits.^20^^,^^21^ While most prior studies have focused on candidate regions such as the *ADIPOQ* promoter, only one epigenome-wide association study (EWAS) has been reported. This EWAS identified one CpG site at suggestive significance in *CPT1A* in a cohort of European and African Americans residing in the United States.^22^ Notably, *CPT1A* has also been detected in obesity and T2D EWAS, suggesting a possible downstream or confounded signal.^23^ Thus far, there is a paucity of EWAS of adiponectin. Adiponectin levels vary across populations, and several studies have shown that individuals of African ancestry, including African Americans and West Africans, tend to have lower adiponectin levels even after adjusting for fat mass, suggesting that other biological mechanisms may be involved.^24^, ^25^, ^26^ Epigenetics provides an attractive framework for exploring these mechanisms, as it captures the influence of both genetic variation and environmental exposures.

Here, we conducted an EWAS of adiponectin levels in two cohorts of West Africans residing in both Africa and Europe. This study aims to identify epigenetic loci associated with adiponectin regulation and to provide insights into its role in cardiometabolic health, advancing our understanding of adiponectin biology in diverse environmental and genetic contexts.

## Methods

### Study population

This analysis was conducted using data from two cohorts: baseline data from the Research on Obesity and Diabetes among African Migrants Prospective (RODAM-Pros) cohort study and data from the Africa America Diabetes Mellitus (AADM) study.

#### RODAM-Pros study

The RODAM-Pros cohort was designed as a population-based study and recruited a representative sample of Ghanaians across distinct environmental and migration contexts (rural, urban, and European settings), thereby capturing broad variability in lifestyle and metabolic exposures. Details on the RODAM-Pros study design have been published previously.^27^^,^^28^ At baseline, 6385 participants aged 18 years or older, born in Ghana to at least one Ghanaian parent, were recruited.

For this analysis, we used a subset of RODAM participants with available DNA methylation data (n = 736). This subset was selected based on a case–control design, comprising approximately 300 untreated diabetic cases, 300 non-diabetic controls, and 135 non-diabetic, non-obese controls. The sample size was originally calculated to provide 80% power to detect a 5% difference in DNA methylation between diabetic cases and controls. After excluding individuals who failed quality control (n = 23), lacked adiponectin measurements (n = 395), or had outlier adiponectin values, defined as ±3 SD from the mean of log-transformed adiponectin levels (n = 3),^28^^,^^29^ the final RODAM-Pros study analytic sample for the present analyses comprised 315 individuals.

Although this analytic subset is not fully representative of the original RODAM-Pros population and the Ghanaian population, such lack of representativeness is unlikely to bias the adiponectin-methylation associations under study. Moreover, stratified analyses by T2D status and subsequent meta-analyses were used to minimise potential bias related to the enrichment of T2D individuals and enhance generalisability.

#### AADM study

The AADM study, the longest-running genetic epidemiology cohort of T2D in sub-Saharan Africans, was established to investigate diabetes and related metabolic traits and includes both individuals with and without T2D. A total of 615 Nigerian participants from a single site (Ibadan) of the AADM study with available DNA methylation and adiponectin data were included in the present analysis. Details on the design and recruitment of the AADM study has been described in previous publications.^30^^,^^31^

After excluding individuals who failed quality control (n = 9), lacked adiponectin measurements (n = 4), or had outlier adiponectin values defined as ±3 SD from the mean of log-transformed adiponectin levels (n = 9), the final AADM sample comprised 593 individuals. Similar quality control procedures were applied as in the RODAM study, as well as a similar stratification approach to minimise potential bias related to the enrichment of T2D individuals.

### Ethics

Ethical approval for the RODAM-Pros study was granted by the ethics committees of the following institutions: Kwame Nkrumah University of Science & Technology (Ghana: CHRPE/AP/200/12), Amsterdam University Medical Center (Netherlands: W12-062#12.17.0086), Charité University Berlin (Germany: EA1/307/12), and London School of Hygiene & Tropical Medicine (UK: 6208). For the AADM study, ethical approval was obtained from the National Institutes of Health and from the National Health Research Ethics Committee of Nigeria (NHREC).

All participants provided written informed consent before enrolment in the studies. All procedures were conducted in accordance with the ethical standards outlined in the 1964 Declaration of Helsinki and its subsequent amendments, or comparable ethical standards.

### Phenotypic measurements

For both cohorts, demographic and lifestyle information was collected through self-administered or interviewer-administered questionnaires, which included data on age and sex. Participants' height and weight were measured in light clothing and without shoes, to the nearest 0.1 cm and kg, respectively. Body mass index (BMI) was calculated as weight (kg) divided by height squared (m^2^). For physical examinations, participants were instructed to fast overnight. Venous blood samples were collected by trained research staff for biochemical analysis.

T2D status was determined based on the American Diabetes Association (ADA) criteria, which include a fasting plasma glucose level of ≥7.0 mmol/L (126 mg/dL), self-reported T2D following physician diagnosis, or use of glucose-lowering medications confirmed by clinical records. Analyses were conducted using complete cases only, after excluding participants with missing data on key variables (adiponectin levels, covariates, or methylation values) to ensure consistency across models.

### Measurement of adiponectin

For RODAM-Pros study participants, circulating adiponectin levels were measured using enzyme-linked immunosorbent assay (ELISA) kits (Crystal Chem, Elk Grove, Illinois, USA). According to the manufacturer, this assay has a dynamic range of 2–100 ng/mL and a sensitivity of 0.3 ng/mL.

In the AADM study, adiponectin levels were measured using multiplex bead-based flow cytometric immunoassays, which use dyed microspheres linked with monoclonal antibodies specific for proteins in the plex, according to the manufacturer's instructions (Bioplex, Bio-Rad, Inc, Hercules, CA, USA). Adiponectin was included in the Bio-Plex Pro Human Diabetes Adipsin and Adiponectin Assays (171A7002M). Circulating levels were determined using a Bio-Plex 200 array reader (Luminex, Austin, TX), and values were calculated using a standard curve with the manufacturer-provided Bio-Plex Manager Software.

The Bio-Plex assay has a working range of 0.056–918.7 ng/mL and a lower limit of detection (LOD) of 0.031 ng/mL. Both the ELISA and multiplex assay measure total adiponectin rather than specific isoforms. Their overlapping dynamic ranges support comparability of results across cohorts. Adiponectin levels were natural log-transformed in all analyses.

### DNA methylation profiling, processing and quality control

DNA methylation profiling was performed by Source BioScience, Nottingham, UK, for the RODAM cohort and by the University of Colorado, USA, for the AADM cohort. The Zymo EZ DNA Methylation™ kit was used for bisulphite DNA treatment. Following a conversion quality check using high-resolution melting analysis, DNA methylation profiles for RODAM-Pros were determined using the Illumina HumanMethylation450 BeadChip, while the Illumina EPIC array was used for AADM samples. DNA methylation levels were quantified for approximately 485,000 CpG sites in RODAM-Pros and over 850,000 CpG sites in AADM by measuring the intensities of methylated and unmethylated probes. The EPIC array includes over 90% of CpG sites present on the 450K array, allowing substantial overlap and comparability between platforms.

Quality control was performed using the *MethylAID* package (version 1.38.0) in the RODAM study and using the *SeSAMe* R/Bioconductor package on AADM study samples. Beta values were calculated to represent the proportion of methylation at each site, ranging from 0 (unmethylated) to 1 (fully methylated). Using R statistical software (version 4.2.0), Beta values were obtained using the getBeta function from the minfi package (version 1.42.0). Beta values were normalised to remove unwanted technical variation. M values, calculated as the log_2_ ratio of Beta values, were subsequently used in statistical analyses as recommended by Du et al.^32^ After the removal of probes annotated to the X and Y chromosomes and cross-reactive probes, a set of 415,405 CpG sites remained for the RODAM study samples and 784,765 CpG sites for AADM study samples. An estimation of cell type distribution was done following the method described by Houseman et al.^33^

### RNA sequencing

RNA sequencing (RNA-seq) data of primary tissues obtained from a subset of AADM participants who also had DNA methylation data were used to investigate whether methylation at identified loci was associated with gene expression in metabolically relevant tissues. Although DNA methylation was measured in peripheral blood for both cohorts, RNA-seq data were available in AADM for blood (n = 77), subcutaneous adipose tissue (SAT; n = 49), and skeletal muscle (n = 55), enabling exploration of tissue-specific expression patterns. RNA sequencing was performed by the NIH Intramural Sequencing Center (NISC) following standard protocols. RNA was extracted from blood samples using the E.Z.N.A.® Blood RNA kit (www.omegabiotek.com) following the manufacturer's instructions. Briefly, cells were collected by centrifugation. Cells were washed and lysed under an optimised buffer containing Proteinase K. Samples were transferred to a Homogeniser Mini Column to remove cell debris and other particulates. Samples were passed through the HiBind® RNA Mini Column, which binds RNA. Genomic DNA was removed from the column by digestion with DNase I. After washing, purified RNA was eluted with RNase-free water.

For adipose tissue and skeletal muscle, RNA was extracted using the E.Z.N.A.® Total RNA Kit II (www.omegabiotek.com). Tissues were first homogenised with RNA-Solv® reagent that inactivates RNases. After adding chloroform, a homogenate was separated into aqueous and organic phases by centrifugation. The aqueous phase, which contains RNA, was passed through a HiBind® RNA Mini column to extract RNA. After washing, RNA was eluted in DEPC water. All RNA samples were quantified using Nanodrop (www.thermofisher.com) and quality was assessed by a TapeStation System (www.agilent.com).

Sequencing libraries were constructed from 200 to 1000 ng total RNA using “TruSeq Stranded Total RNA Sample Prep Guide with Ribo Zero Gold/Globin” (Illumina, Inc.) used according to manufacturer's instructions. Libraries were tagged with unique dual indexes. Amplification was performed using 10–16 cycles based on input amount. Libraries were pooled in equimolar amounts for sequencing. The pooled libraries were sequenced on an S4 flow cell on a NovaSeq 6000 DNA Sequencer (Illumina, Inc.) to generate a minimum of 50M 150b paired-end reads per library.

Reads were aligned to the GRCh38 reference genome using RSEM v1.3.3 and BOWTIE2 v2.5.3 with standard parameters.^34^^,^^35^ Gene expression levels were quantified using RSEM and reported as transcripts per million (TPM).

Expression quantitative trait methylation (eQTM) analyses were then performed using the Matrix eQTL package to assess associations between methylation M-values and gene expression levels. Analyses were conducted separately for blood, subcutaneous adipose tissue (SAT), and skeletal muscle, adjusting for age, sex, and medication use. This approach enabled evaluation of tissue-specific relationships between DNA methylation at significant CpG sites (identified from blood-based EWAS) and gene expression in metabolically relevant tissues.

### Statistics

#### Study cohort characteristics

Statistical analyses were performed using the R statistical computing environment. Study cohort characteristics were summarised as proportions for categorical variables, as means (with standard deviations) for normally distributed continuous variables, and as medians (with interquartile range) for non-normally distributed continuous variables. Normality of continuous variables was assessed using visual inspection of histograms and Q–Q plots, supported by descriptive statistics generated in R (including skewness and kurtosis measures) to guide the appropriate choice between parametric and non-parametric summaries. Analyses were stratified by T2D case/control status due to the established relationship between T2D and adiponectin levels, as well as the enrichment of T2D cases in both cohorts (Table 1).

**Table 1:** Characteristics of study population.

	AADM (N = 593)		RODAM-Pros (N = 315)	
	Non-T2D (n = 316)	T2D (n = 277)	Non-T2D (n = 203)	T2D (n = 112)
Age	55 ± 12	60 ± 10	51 ± 9	53 ± 10
Female	242 (76.6)	221 (79.8)	125 (61.6)	64 (57.1)
Male	74 (23.4)	56 (20.2)	78 (38.4)	48 (42.9)
Site				
Urban Nigerians, %	316 (100)	277 (100)	–	–
Rural Ghanaians, %	–	–	53 (58.24)	38 (41.76)
Urban Ghanaians, %	–	–	63 (64.29)	35 (35.71)
Amsterdam Ghanaians, %	–	–	87 (69.05)	39 (30.95)
BMI, kg/m^2^	30.8 (26.3–35.1)	31.0 (27.0–36.0)	24.2 (21.6–29.8)	26.2 (22.6–30.1)
Adiponectin, μg/mL	6.10 (4.02–10.10)	6.24 (3.47–11.40)	7.41 (4.42–11.21)	5.33 (3.07–9.00)
Immune cells				
CD8+ T cells, proportion	0.15 ± 0.05	0.15 ± 0.05	0.12 ± 0.05	0.11 ± 0.05
CD4+ T cells, proportion	0.20 ± 0.06	0.19 ± 0.06	0.19 ± 0.06	0.17 ± 0.05
Natural killer (NK) cells, proportion	0.06 ± 0.03	0.05 ± 0.03	0.11 ± 0.06	0.11 ± 0.06
B cells, proportion	0.09 ± 0.05	0.08 ± 0.03	0.11 ± 0.03	0.11 ± 0.04
Monocytes, proportion	0.08 ± 0.02	0.08 ± 0.02	0.08 ± 0.03	0.08 ± 0.02
Granulocytes, proportion	0.41 ± 0.10	0.44 ± 0.10	0.44 ± 0.09	0.46 ± 0.09

Data are presented as mean ± SD, median (IQR), or n (%). Immune cell estimates are shown as proportions (0–1) based on Houseman deconvolution and may not sum to 1 due to rounding.

#### Differentially methylated positions (DMPs)

Differentially Methylated Positions (DMPs) were identified using the *limma* package (version 3.52.4) in each cohort separately. Linear regression models were employed to assess associations between DNA methylation (dependent variable) and log-transformed adiponectin levels (independent variable) using the *minfi* package. Given the established relationship between adiposity and adiponectin, BMI was included as a covariate in all models rather than as a stratification factor to preserve statistical power and control for confounding.

For both cohorts, models were adjusted for age, sex, BMI, and estimated cell counts. Estimated immune cell proportions (CD8+ T cells, CD4+ T cells, natural killer cells, B cells, monocytes, and granulocytes) were derived using the Houseman algorithm^33^ implemented in the *minfi* package. Covariates were selected based on principal component analysis (PCA), which indicated these factors were the primary contributors to variation in methylation data. Both male and female participants were included in all analyses, and sex was included as a covariate to control for sex-related differences in adiponectin levels and DNA methylation. Models for RODAM-Pros study data were additionally adjusted for recruitment site, hybridisation batch and position on the plate, which were previously identified as relevant technical covariates for this cohort.^36^ Given the strong influence of T2D on both adiponectin levels and DNA methylation,^36^ all DMP analyses were stratified by T2D status to minimise confounding. Although BMI and adiponectin levels are biologically interrelated, we included BMI as a covariate to isolate methylation associations uniquely attributable to adiponectin levels.

Meta-analyses were subsequently conducted on the 409,033 CpG sites shared between the 450K (RODAM-Pros) and EPIC (AADM) arrays, combining participants with and without T2D from both cohorts to identify DMPs associated with adiponectin in West Africans across diabetes status. To explore differences in DNA methylation patterns between T2D cases and controls, and between Ghanaians (RODAM-Pros study) and Nigerians (AADM study), additional stratified meta-analyses were conducted for T2D cases and controls separately, and for Ghanaians and Nigerians separately.

Meta-analyses were performed using *METAL software*,^37^ employing an inverse-variance weighted fixed-effects model. A fixed-effects approach was selected because only two cohorts were included, making random-effects modelling statistically unreliable and potentially unstable. This model assumes a common underlying effect across studies while accounting for sampling variance, which aligns with our goal of identifying shared methylation–adiponectin associations among West Africans. Between-cohort heterogeneity was formally evaluated using Cochran's Q test and the I^2^ statistic to ensure robustness of combined estimates. QQ-plots demonstrated well-controlled inflation (Supplementary Figures S1, S2 and S3). Statistical significance was determined using P values derived from M values, with multiple testing corrected using the Benjamini-Hochberg false discovery rate (FDR). CpG sites reaching an FDR of <0.05 were considered genome-wide significant. While M-values were used for fitting the EWAS models, beta-values were employed for visualisation to enhance interpretability. Z-scores were generated as part of the METAL meta-analysis output and represent the direction and strength of association across cohorts, standardised by standard error.^37^ These values were reported for genome-wide significant CpG sites to facilitate comparison across loci.

#### Differentially methylated regions (DMRs)

DMRs were identified using the *bumphunter* function from the *minfi* package in RODAM-Pros and AADM separately. Regions were defined as three or more adjacent methylation probes with an effect size cut-off of 0.1. Statistical significance was determined using a family-wise error rate (FWER) threshold of <0.25. This threshold was chosen following recommendations for the *bumphunter* algorithm, as region-level permutation tests are inherently conservative; FWER <0.25 is widely used in EWAS to retain adequate power for region detection.^38^

To increase power for region-level analysis and overcome the requirement of individual-level data in *bumphunter,* we additionally applied *DMRff*^39^ to meta-analysed EWAS summary statistics. *DMRff* identifies regions of spatially correlated methylation changes using combined regression coefficients and standard errors across studies. The analysis was performed using METAL summary statistics from both cohorts, with genomic coordinates aligned to GRCh37/hg19. Adjacent CpGs (<500 bp apart) with consistent direction of effect were grouped into regions, and FDR correction was applied to identify significant DMRs (FDR <0.05). Gene annotation was based on UCSC (GRCh37/hg19).

### In-silico functional annotation to assess biological relevance

To assess the biological significance of the identified DMPs and DMRs, we conducted a multi-step in-silico functional validation including methylation-expression correlation (eQTM), pathway enrichment, and regulatory feature enrichment analyses. This was followed by gene annotation and literature-based functional characterisation.

#### Expression quantitative trait methylation (eQTM) analysis

To explore the relationship between DNA methylation and gene expression, expression quantitative trait methylation (eQTM) also known as methylation-expression associations-analyses were performed using the subset of AADM data with expression data available. The Matrix eQTL tool was used to identify associations between methylation M values and gene expression in blood, subcutaneous adipose tissue (SAT), and skeletal muscle tissue. Both cis- and trans-eQTMs were analysed separately for each tissue type, adjusting for age, sex, and T2D medication use. All eQTM models were adjusted for these covariates to minimise confounding, consistent with established practice for cross-tissue methylation-expression analyses. These covariates were selected to harmonise adjustment across blood, SAT, and skeletal muscle datasets while accounting for key demographic and clinical factors relevant to both DNA methylation and gene expression variation. An FDR threshold of <0.05 was applied to identify statistically significant eQTMs.

We additionally queried methylation-expression associations for the top DMPs using public eQTM resources through the iMETHYL database (http://imethyl.iwate-megabank.org).^40^ IMETHYL provides whole genome DNA methylation, and whole-transcriptome data for normal CD4 + T-lymphocytes, monocytes, and neutrophils collected from approximately 100 healthy subjects.

#### Pathway enrichment analyses

Pathway enrichment analyses were performed using the *missMethyl* and *clusterProfiler* R packages. missMethyl accounts for bias from unequal probe representation per gene on Illumina arrays. Analyses were based on CpGs ranked by EWAS p-values in each cohort, selecting the top 1000 and 5000 CpGs as input. Gene Ontology (GO) and Kyoto Encyclopedia of Genes and Genomes (KEGG) pathways were evaluated using the *gometh* () function with array.type = “450K” and prior probability correction enabled. *clusterProfiler* was used in parallel to confirm enrichment patterns via *enrichGO()* and *enrichKEGG()* functions. Pathways were considered significant at FDR <0.05. As a sensitivity analysis, enrichment was repeated using all CpGs with FDR <0.4.

#### Chromatin state and regulatory feature enrichment

We used eFORGE (https://eforge.altiusinstitute.org) to test whether adiponectin-associated CpGs were enriched for regulatory genomic features. Analyses included CpGs with FDR <0.2 and FDR <0.4 from the meta-analysis. Enrichment was tested across datasets from the Roadmap Epigenomics and ENCODE projects, including DNase I hypersensitivity sites (DHS), consolidated 15-state chromatin models, and histone modification marks (H3K4me1, H3K4me3, H3K27ac). Enrichment was considered significant at permutation-based FDR <0.05.

#### Literature-based functional characterisation

Gene annotation and functional characterisation were conducted using GeneCards (Weizmann Institute of Science),^41^ NCBI Gene (formerly PubMed Gene, NIH),^42^ and the EWAS Atlas (National Genomics Data Center).^43^ Next, a comprehensive literature review was carried out to evaluate the relevance of annotated genes to adiponectin and related metabolic processes. Additionally, publicly available databases such as the GWAS Catalogue (NHGRI-EBI), dbSNP, and EWAS Catalogue were queried to determine whether the identified DMPs and DMRs had prior associations with adiponectin or related cardiometabolic traits. These databases focused specifically on trait-association data, where cg IDs and/or annotated gene names were cross-referenced to identify prior associations.

### Role of the funders

The funders had no role in the study design; collection, analysis, or interpretation of data; writing of the report; or the decision to submit the manuscript for publication.

### Sensitivity analysis

#### Geographical site-specific variation

To assess the potential impact of geographical environment on our identified CpG sites, a sensitivity analysis was conducted to examine the distribution of DNA methylation levels across three geographical locations in the RODAM cohort: Rural Ghana, Urban Ghana, and Amsterdam. For each site, methylation levels were visualised using violin plots, and statistical differences in the distribution of methylation levels across the three sites were evaluated using the Kruskal–Wallis test. This approach enabled an assessment of whether environmental factors that may differ across geographic locations contributed to variation in DNA methylation of sites relevant to adiponectin.

#### Assessment of between-cohort heterogeneity

Between-cohort heterogeneity was quantified using Cochran's Q test and the I^2^ statistic implemented in METAL. I^2^ values greater than 50% and Q-test p-values below 0.05 were considered indicative of significant heterogeneity, suggesting potential cohort-specific effects.

#### Model collinearity assessment

To evaluate potential multicollinearity among covariates included in the EWAS models, pairwise Pearson correlations and variance inflation factors (VIFs) were computed separately for the RODAM and AADM cohorts. Each model included age, sex, body mass index (BMI), recruitment site, hybridisation batch, position on the plate, and estimated immune cell proportions (CD8^+^ T cells, CD4^+^ T cells, natural killer cells, B cells, monocytes, and granulocytes) derived using the Houseman algorithm implemented in *minfi.* VIFs were calculated using the car package in R based on linear models with log-transformed adiponectin as the dependent variable, consistent with the EWAS specification. Adjusted VIFs were expressed as GVIF^1^∕(^2^ × Df), where Df denotes the degrees of freedom. Values below 5 were interpreted as indicating no collinearity and values below 10 as acceptable.

## Results

### Cohort characteristics

The total study cohort comprised 908 West Africans, including 315 Ghanaians from the RODAM-Pros study and 593 Nigerians from the AADM study. Participants with T2D were older than non-T2D individuals in both cohorts (e.g., 53 ± 10 vs. 51 ± 9 years in RODAM-Pros; 60 ± 10 vs. 55 ± 12 years in AADM), and women were more prevalent in AADM (77–80%) than in RODAM-Pros (57–62%) (Table 1).

BMI was higher in AADM (30.8–31.0 kg/m^2^) than in RODAM-Pros (24.2–26.2 kg/m^2^), consistent with higher adiposity among AADM participants. Median adiponectin levels were similar across cohorts, though lower in T2D vs. non-T2D participants within RODAM-Pros (5.33 vs. 7.41 μg/mL). This may reflect paradoxical hyperadiponectinaemia, where higher adiponectin is observed despite obesity or insulin resistance.

RODAM-Pros participants were recruited from rural and urban Ghana and Amsterdam, while all AADM participants were urban Nigerians. Immune cell proportions showed minimal variation across groups (Table 1).

### Differentially Methylated Positions (DMPs)

#### Adiponectin DMPs in West Africans

**Fig. 1:**
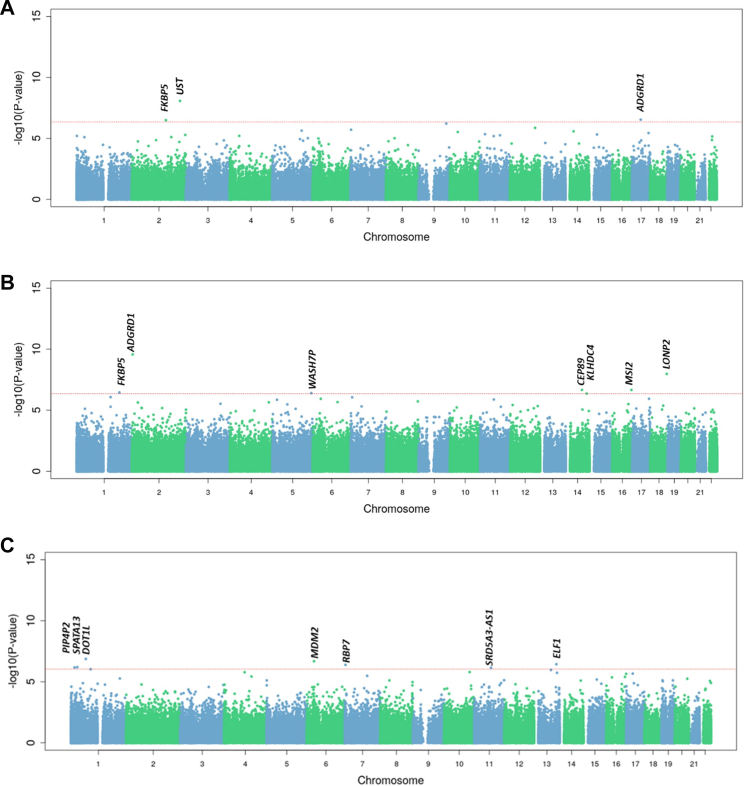
Manhattan plot of epigenome-wide association analysis for circulating adiponectin in A) All West Africans combined (n = 908), B) Nigerians from the AADM cohort (n = 593), C) Ghanaians from the RODAM-Pros cohort (n = 315). *The red line indicates an FDR < 0.05.*

A meta-analysis of all 908 West African participants identified three CpGs significantly associated with adiponectin levels across T2D status, combining participants with and without diabetes (Fig. 1A; Table 2). These included cg03546163, annotated to *FKBP5* (Z-score = −5.76, FDR = 0.0032, p < 0.001), cg02561343, annotated to *UST* (Z-score = 5.11, FDR = 0.0415, p < 0.001), and cg23969380, annotated to *ADGRD1* (Z-score = 5.13, FDR = 0.0415, p < 0.001). Higher adiponectin levels were associated with lower DNA methylation at the *FKBP5* CpG site, while lower methylation at CpGs annotated to *UST* and *ADGRD1* was associated with lower adiponectin levels. The direction of effect was consistent across the individual populations (Table 2).

**Table 2:** Epigenome-wide significant differentially methylated positions (DMPs) associated with circulating adiponectin.

CpG ID	Chr:Pos (GRCh37)	Nearest gene (Symbol)	Gene feature	All West Africans		AADM		RODAM	
				Z-score (FDR)	Raw p-val	Z-score (FDR)	Raw p-val	Z-score (FDR)	Raw p-val
cg03546163	6:35654363	*FKBP5*	5′UTR	**−5.76 (0.0032)**	***<0.001***	**−6.32 (0.0002)**	***2.71 × 10*^*−*^*^10^***	−1.23 (0.8914)	***1.35 × 10*^*−*^*^7^***
cg02561343	6:149291652	*UST*	Within body	**5.11 (0.0415)**	***<0.001***	4.072 (0.2774)	*4.67E-05*	3.05 (0.5477)	*0.002282*
cg23969380	12:131708174	*ADGRD1*	Intergenic	**5.13 (0.0415)**	***<0.001***	**5.72 (0.0041)**	***1.06 × 10*^*−*^*^8^***	0.92 (0.9291)	*0.358*
cg19208107	1:221000000	*WASH7P*	Intergenic	–	–	**−5.18 (0.0429)**	**2.18 × 10^−7^**	–	–
cg21378860	19:33371965	*CEP89*	Within body	–	–	**−5.18 (0.0429)**	**2.19 × 10^−7^**	–	–
cg00756062	16:87695845	*KLHDC4*	Within body	4.52 (0.1500)	6.32E-06	**5.09 (0.0486)**	***3.52 × 10*^*−*^*^7^***	0.75 (0.9454)	0.4528
cg07768696	17:55676682	*MSI2*	Within body	–	–	**5.07 (0.0486)**	**3.98 × 10^−7^**	–	–
cg19772756	16:48201510	*LONP2*	Within body	–	–	**−5.05 (0.0486)**	**4.34 × 10^−7^**	–	–
cg00778059	13:24734742	*SPATA13*	TSS200	−3.22 (0.5900)	0.00128	−0.12 (0.9977)	0.9074	**−5.27 (0.0439)**	***1.35 × 10*^*−*^*^7^***
cg00940812	8:91960827	*PIP4P2*	Within body	−2.68 (0.6889)	0.007355	0.34 (0.9940)	0.7309	**−4.97 (0.0439)**	***6.80 × 10*^*−*^*^7^***
cg01971579	19:2164932	*DOT1L*	Within body	−2.71 (0.6832)	0.006728	0.33 (0.9940)	0.7393	**−4.98 (0.0439)**	***6.35 × 10*^*−*^*^7^***
cg08797454	12:69202718	*MDM2*	Within body	−3.56 (0.4926)	0.000377	−0.53 (0.9902)	0.5964	**−5.19 (0.0439)**	***2.08 × 10*^*−*^*^7^***
cg10796749	1:10056837	*RBP7*	TSS1500	4.76 (0.1271)	1.95E-06	2.15 (0.8138)	0.03163	**5.06 (0.0439)**	**4.13 × 10^−7^**
cg16225703	4:62383028	*SRD5A3-AS1*	Within body	–	–	–	**–**	**−4.96 (0.0439)**	**7.17 × 10^−7^**
cg18456803	13:41593519	*ELF1*	TSS200	−4.17 (0.2792)	3.08E-05	−1.41 (0.9395)	0.1572	**−5.09 (0.0439)**	**3.66 × 10^−7^**

Meta-analysis results for all West Africans combined (n = 908) include Z-scores, raw p-values, and FDR values derived using METAL. Cohort-specific EWAS results are shown for the AADM study (n = 593) and the RODAM-Pros study (n = 315). “–” indicates that the CpG was not available in a given cohort due to array coverage differences between the EPIC and 450K platforms. Bolded values denote CpGs reaching epigenome-wide significance at FDR <0.05. Raw p-values are reported exactly as produced by METAL; for readability, values < 0.001 are displayed as “< 0.001.”

#### Adiponectin DMPs in AADM

In AADM participants, seven CpGs were associated with adiponectin levels (Fig. 1B and Table 2). Two CpGs overlapped with those identified in the combined West African models. The top signal, cg03546163 (*FKBP5*) had a stronger effect size and lower p-value (Z-score = −6.315, FDR = 0.0002, p = 2.71 × 10^−10^) in AADM than in the combined West African analysis. The CpG cg23969380 (*ADGRD1*) showed a consistent positive association with adiponectin levels, as also observed in the combined West African model (Z-score = 5.721, FDR = 0.00417, p = 1.06 × 10^−8^). Additional genome-wide significant CpGs were annotated to *WASH7P* (cg19208107; Z-score = −5.183, FDR = 0.0429, p = 2.18 × 10^−7^), *CEP89* (cg21378860; Z-score = −5.183, FDR = 0.04295, p = 2.19 × 10^−7^), *KLHDC4* (cg00756062; Z-score = 5.093, FDR = 0.0486, p = 3.52 × 10^−7^), *MSI2* (cg07768696; Z-score = 5.070, FDR = 0.0486, p = 3.98 × 10^−7^), and *LONP2* (cg19772756; Z-score = −5.053, FDR = 0.0486, p = 4.34 × 10^−7^). The associations were directionally consistent with those observed in the combined West African analyses, with *KLHDC4* and *MSI2* showing positive effects, and the remaining CpGs showing negative associations with adiponectin levels (Table 2).

#### Adiponectin DMPs in RODAM-Pros

Among RODAM participants, a different set of seven CpGs were significantly associated with adiponectin levels (Fig. 1C and Table 2). The top CpG was cg00778059, annotated to *SPATA13* (Z-score = −5.272, FDR = 0.04399, p = 1.35 × 10^−7^), followed by cg00940812 (*PIP4P2*; Z-score = −4.967, FDR = 0.0439, p = 6.80 × 10^−7^), cg01971579 (*DOT1L*; Z-score = −4.980, FDR = 0.0439, p = 6.35 × 10^−7^), cg08797454 (*MDM2*; Z-score = −5.192, FDR = 0.0439, p = 2.08 × 10^−7^), cg10796749 (*RBP7*; Z-score = 5.063, FDR = 0.0439, p = 4.13 × 10^−7^), cg16225703 (*SRD5A3-AS1*; Z-score = −4.957, FDR = 0.0439, p = 7.17 × 10^−7^), and cg18456803 (*ELF1*; Z-score = −5.086, FDR = 0.0439, p = 3.66 × 10^−7^). Except for *RBP7*, which showed a positive association, all other DMPs demonstrated negative associations with adiponectin levels. These associations were consistent across individuals with and without T2D within the Ghanaian cohort (Table 2).

#### Adiponectin DMPs by T2D status

Meta-analyses conducted combining all West Africans stratified by T2D status did not identify any CpGs meeting the epigenome-wide significance threshold (Supplementary Figure S4). The three CpGs identified in the main non-stratified West African analyses (cg03546163, cg02561343, and cg23969380) showed FDR values of 0.72, 0.66, and 0.81 in T2D cases, compared with 0.18, 0.49, and 0.33 in T2D controls, respectively. This suggests that while no CpGs reached significance within the T2D subgroups, the main model identified CpGs may be more driven by the T2D control subgroup than by the T2D cases subgroup. When stratified by T2D status and by cohort, a few significant DMPs were observed, though none overlapped with those identified in the main meta-analyses (details are provided in Supplementary Results S1, Supplementary Figures S5 and S6).

### Differentially methylated regions

Among AADM, one significant DMR was detected on chromosome 12 (12:739,953–740,338), annotated to the *NINJ2* gene, showing a negative association with adiponectin (effect size = −0.311, FDR = 0.008). No overlap was observed between the significant DMPs and the identified DMRs (Table 3).

**Table 3:** Differentially methylated regions (DMRs) associated with circulating adiponectin in Nigerians and Ghanaians.

Chr	Start	End	CpGs	Gene	Effect size	FWER	Population
chr12	739,953	740,338	1	*NINJ2*	−0.311	0.008	Nigerians
chr6	30,038,955	30,039,206	15	*RNF39*	0.525	0.062	Ghanaians
chr6	30,039,374	30,039,548	12	*RNF39*	0.575	0.080	Ghanaians

**Chr**: The chromosome number on which the DMR is located. **Start/End**: The start and end positions (in CRCh37) of the DMR. **CpGs**: The number of CpGs identified within the region. **Gene**: The gene(s) annotated to the DMR. **Effect size**: The effect size of the methylation change. **FWER**: family-wise error rate.

Two significant DMRs were identified among Ghanaians from the RODAM-Pros study. The first DMR, spanning 15 CpGs, was located on chromosome 6 (6:30,038,955–30,039,206) and showed a positive association with adiponectin (effect size = 0.525, FDR = 0.062). The second DMR, encompassing 12 CpGs, was located nearby (6:30,039,374–30,039,548) and was similarly positively associated (effect size = 0.575, FDR = 0.080). Both DMRs were annotated to the *RNF39* gene (Table 3).

In the meta-analysis of regional methylation patterns across RODAM and AADM using the DMRff approach, 1410 significant DMRs (FDR <0.05) were identified (Supplementary Table S1). The top 10 regions are presented in Table 4. The strongest DMR (FDR <0.001) was located on chromosome 1, annotated to the *TP73/WDR8* locus (β = −0.0069, SE = 0.0002), followed by regions annotated to *DIO3/MIR1247* (chr14, β = −0.0074, FDR = 0.000), *EYA4* (chr6, β = −0.0050, FDR = 0.000), *HOXD8* (chr2, β = −0.0107, FDR = 0.000), and *GULP1* (chr2, β = −0.0057, FDR = 0.000). Additional significant regions were located at *EDNRB* (chr13, β = −0.0029, FDR = 0.000), *EPB41L3* (chr18, β = −0.0071, FDR = 0.001), *FOXG1* (chr14, β = −0.0042, FDR = 0.001), *GABRA2* (chr4, β = −0.0036, FDR = 0.001), and *NKX6-2* (chr10, β = −0.0060, FDR = 0.002). All top DMRs showed consistent hypomethylation across CpGs (β range = −0.011 to −0.004). Several of these genes including *TP73, EYA4, GULP1*, and *FOXG1* are involved in transcriptional control, oxidative stress response, and metabolic signalling, supporting their potential role in adiponectin regulation. The regional clustering of DMRs across endocrine, neuroendocrine, and metabolic loci highlights coordinated epigenetic mechanisms underlying adiponectin-related pathways in West Africans.

**Table 4:** Top 10 differentially methylated regions (DMRs) identified using DMRff meta-analysis.

Chr	Start	End	n (CpGs)	β	SE	FDR	Annotated genes
chr1	3,567,163	3,568,245	19	*−0.0069*	0.0002	0.00E + 00	*TP73, WDR8*
chr14	102,026,230	102,027,172	13	*−0.0074*	0.0002	5.14E-275	*DIO3, MIR1247*
chr6	133,562,087	133,562,492	18	*−0.0050*	0.0001	2.51E-252	*EYA4*
chr2	176,994,362	176,995,088	8	*−0.0107*	0.0003	1.37E-238	*HOXD8*
chr2	189,156,425	189,157,566	14	*−0.0057*	0.0002	6.88E-238	*GULP1*
chr13	78,493,100	78,493,590	19	*−0.0029*	0.0001	4.76E-227	*EDNRB*
chr18	5,543,548	5,544,231	11	*−0.0071*	0.0002	8.50E-226	*EPB41L3*
chr14	29,234,981	29,236,476	19	*−0.0042*	0.0001	1.16E-210	*FOXG1*
chr4	46,391,259	46,392,526	15	*−0.0036*	0.0001	1.31E-208	*GABRA2*
chr10	134,599,841	134,600,701	17	*−0.0060*	0.00020	1.64E-207	*NKX6-2*

Top 10 DMRs associated with circulating adiponectin levels identified using DMRff meta-analysis with regions defined as clusters of CpGs (<500 bp apart) exhibiting consistent direction of effect across RODAM and AADM cohorts. Coordinates are based on genome build GRCh37/hg19. Beta values represent the direction and magnitude of association between regional methylation and adiponectin levels. Abbreviations: SE, standard error; FDR, false discovery rate.

### Biological relevance

In the pathway analysis using the KEGG and EWAS atlas, there was no identified enrichment in gene ontology.

The significant DMRs that were identified include regions annotated to *NINJ2*, a gene involved in adipocyte differentiation and insulin signalling and *RNF39*, a gene involved in metabolic regulation.

The top regions from the *DMRff* meta-analysis mapped to genes such as *TP73-WDR8, TNFRSF18*, *AJAP1*, *CAMTA1*, and *RBP7*, which are implicated in transcriptional control, immune modulation, and lipid and glucose metabolism. The *TP73-WDR8* locus has been linked to apoptosis and cellular stress responses, while *TNFRSF18* participates in TNF receptor-mediated signalling, and *RBP7* regulates retinoid transport and lipid homoeostasis. Collectively, these DMRs highlight pathways related to immune-metabolic regulation consistent with adiponectin's known anti-inflammatory and insulin-sensitising functions.

We identified eQTMs for several of the epigenome-wide significant CpGs across three tissues; blood, SAT, and skeletal muscle using RNA-seq data from AADM participants who also had blood-based methylation data. In SAT, higher methylation at cg03546163 (*FKBP5*) was associated with reduced expression of *PLA2G12B*, a gene involved in fatty acid and glucose metabolism. Similarly, higher methylation at cg02561343 (*UST*) was associated with reduced expression of *PSMD8* and *TECR*, involved in proteasome function and lipid metabolism, respectively. In blood, higher methylation at cg23969380 (*ADGRD1*) was associated with reduced expression of *HIGD2AP1*, which plays a role in cellular adaptation to hypoxia. No significant eQTMs were observed in skeletal muscle or blood (Table 5).

**Table 5:** Expression quantitative trait methylations (eQTMs) for adiponectin differentially methylated positions in West Africans combined.

CpG	Gene	CpG chr:pos (GRCh37)	Gene chr:pos (GRCh37)	Effect Size (Beta)	p-value	FDR	Tissue
cg03546163	*PLA2G12B*	Chr6: 35,654,363	chr10:74,694,520–74,714,564	−0.0392	8.19E-06	0.047	SAT
cg02561343	*PSMD8*	Chr6: 149,291,652	chr19:38,865,211–38,874,464	−11.849	4.20E-06	0.029	SAT
cg02561343	*TECR*	Chr6: 149,291,652	chr19:14,640,372–14,676,792	−9.477	4.23E-06	0.029	SAT
cg23969380	*HIGD2AP1*	Chr12:131,708,174	chr2:232,301,820–232,302,132	−0.095	1.91E-05	0.024	Blood

CpG = identifier of the differentially methylation position identified in EWAS, Gene = gene whose expression is associated with the methylation level at the CpG, SAT = Subcutaneous Adipose Tissue.

### In-silico functional annotation to assess biological relevance

Methylation-expression correlations provided the first layer of functional insight into the adiponectin-associated CpGs. Integration with the *iMETHYL* database demonstrated that hypermethylation of cg03546163 (*FKBP5*) and cg02561343 (*UST*) was associated with reduced gene expression, consistent with their inverse relationships to circulating adiponectin levels in our EWAS. cg23969380 (*ADGRD1*) showed similar regulation in blood-derived tissues. Cross-referencing with GTEx-based eQTM resources further suggested concordant tissue-specific methylation–expression relationships in adipose tissue, skeletal muscle, and blood, reinforcing the functional relevance of these loci (Table 6).

**Table 6:** Summary of in-silico functional and regulatory enrichment analyses of adiponectin-associated CpGs.

Analysis type	Dataset/Tool	Key findings	Representative pathways/tissues	Top-features (mark/GO term)	Nominal p-value	FDR (q-value)	Interpretation
Pathway enrichment (GO)	missMethyl/clusterProfiler	Enrichment for biological processes related to heart-process, neurogenesis, and synaptic organisation	RODAM: heart contraction; AADM: nervous system development	*GO:0003015 “heart process”, GO:0007399 “neurogenesis”*	<0.05	<0.05	*Adiponectin CpGs map to cardiovascular and neural signalling pathways*
Pathway enrichment (KEGG)	missMethyl/clusterProfiler	Nominal enrichment for metabolic and signalling pathways	Glycolysis/Gluconeogenesis; Neuroactive ligand–receptor interaction	*KEGG:00010, KEGG:04080*	<0.05	<0.1	*Suggests epigenetic involvement in metabolic and synaptic signalling*
Regulatory enrichment (DHS)	Roadmap Epigenomics (erc2-DHS)	CpGs located near DNase I hypersensitive sites	Haematopoietic stem cells, adipose tissue	*DNase I open chromatin regions*	0.034–0.049	1.0	*Open chromatin accessibility in metabolic and immune tissues*
Chromatin-state enrichment	Roadmap Epigenomics (15-state model)	CpGs enriched in enhancer and promoter-flanking states	Haematopoietic stem cells	*Active enhancer (EnhA1), promoter flank (TssAFlnk)*	0.046	1.0	*Preferential localisation within active regulatory regions*
Histone modification enrichment	Roadmap Epigenomics (H3 marks)	Nominal enrichment for active enhancer marks	Embryonic stem cells (E003), haematopoietic stem cells (E051)	*H3K4me1*	0.021–0.054	1.0	*CpGs overlap enhancer histone signatures in developmental and blood cells*
Regulatory enrichment (ENCODE DHS)	ENCODE Consortium	CpGs overlap DNase I hypersensitive regions	Jurkat T cells, CD20^+^ B cells, HMVEC endothelial cells	*DNase I open chromatin*	0.008–0.10	1.0	*Regulatory accessibility in immune and vascular cell types*

Pathway enrichment (GO and KEGG) was assessed using *missMethyl* and *clusterProfiler* with CpGs ranked by EWAS p-values (top 1000 and 5000 inputs). Regulatory enrichment was performed using *eFORGE v2.0* across Roadmap Epigenomics (DNase I hypersensitivity, chromatin 15-state, and H3 histone marks) and ENCODE DNase I datasets. Reported are the most relevant tissues, chromatin marks, and pathways showing nominal or FDR-significant enrichment (p < 0.05). Full enrichment outputs are provided in Supplementary Tables S16–S19.

Pathway enrichment analyses revealed biologically coherent pathways linking adiponectin-associated methylation to cardiometabolic and neuroendocrine regulation. Using *missMethyl,* genes annotated to the top 5000 CpGs in RODAM were enriched for heart process (FDR = 0.0189) and heart contraction (FDR = 0.0241), pointing to cardiovascular function (Supplementary Tables S2 and S3). KEGG pathways showed nominal enrichment for glycolysis/gluconeogenesis and arrhythmogenic right ventricular cardiomyopathy (FDR = 0.83) (Supplementary Tables S4 and S5). In AADM, enrichment was strongest for neuronal and signalling-related processes. Among the top 1000 CpGs, significant GO terms included cell–cell signalling and cartilage condensation (both FDR = 0.0487) (Supplementary Table S6), while the top 5000 CpGs were enriched for nervous system development, neurogenesis, and synaptic membrane organisation (all FDR <0.0001) (Supplementary Table S7). KEGG pathway analysis additionally identified neuroactive ligand–receptor interaction (FDR = 0.0002) and nicotine addiction (FDR = 0.07) (Supplementary Tables S8 and S9). Parallel analyses using *clusterProfiler* corroborated these findings, showing overlapping enrichment in metabolic, muscle contraction, and oxidative phosphorylation pathways in RODAM and neuronal signalling and MAPK cascade regulation in AADM (FDR <0.05) (Supplementary Tables S10–S13). Together, these results suggest that adiponectin-associated methylation signatures converge on cardiometabolic and neuroendocrine pathways, consistent with adiponectin's known systemic roles.

Chromatin state and regulatory feature analyses using *eFORGE* provided further regulatory context. CpGs with FDR <0.2 and < 0.4 were significantly overrepresented in enhancer and flanking active TSS regions (FDR <0.05) across multiple tissues, particularly in adipose, skeletal muscle, and blood, which are key tissues for adiponectin synthesis and metabolic action. These CpGs also overlapped with *H3K27ac* and *H3K4me1* histone marks, supporting their localisation within transcriptionally active regions (Supplementary Tables S16 and S17).

Finally, literature-based gene annotation using GeneCards, NCBI Gene, the EWAS Atlas, and the GWAS Catalogue showed that the genes annotated to the top CpGs have known roles in inflammation, lipid metabolism, stress signalling, and neuroendocrine function. Several CpGs and genes have previously been reported in association with metabolic or cardiometabolic traits, further supporting the biological plausibility of the loci identified in this study.

Collectively, the in-silico analyses demonstrate that adiponectin-associated CpGs exert tissue-relevant transcriptional effects, map to biologically coherent cardiometabolic and neuroendocrine pathways, and localise within transcriptionally active regulatory regions. Together, these findings provide strong evidence for the biological relevance and potential mechanistic role of the identified methylation signals.

### Sensitivity analysis

#### Geographical site-specific variation

**Fig. 2:**
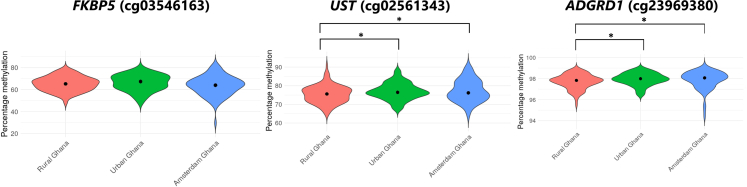
Violin plots of DNA methylation percentages for the three main differentially methylated positions, stratified by location (Rural Ghana, Urban Ghana, and Amsterdam; total n = 315 RODAM-Pros participants). *Asterisks (∗) indicate statistically significant differences across locations (*p < *0.05).*

Comparison of the three significant CpGs from the main DMP analyses in all West Africans revealed minimal site-specific variation in DNA methylation levels, though some differences were observed (Fig. 2). At *FKBP5* (cg03546163), participants from Amsterdam exhibited a wider distribution and a slightly lower median methylation level compared to those from urban and rural Ghana, whose profiles were relatively similar. For *UST* (cg02561343), methylation levels were generally high across all sites (medians between 75 and 80%), with slightly greater variability observed in the Amsterdam group. Methylation at *ADGRD1* (cg23969380) was uniformly high across all locations (medians near 98%), with minimal variation between groups. There were statistically significant differences in methylation levels across sites for both *UST* and *ADGRD1*, but not for *FKBP5* (Fig. 2). These methylation differences for *UST* and *ADGRD1* were primarily driven by contrasts between Rural Ghana and both Urban Ghana and Amsterdam Ghana.

#### Between-cohort heterogeneity

To evaluate the consistency of methylation–adiponectin associations across cohorts, we assessed between-cohort heterogeneity using Cochran's Q test and the I^2^ statistic. The CpGs annotated to *FKBP5* (cg03546163) and *ADGRD1* (cg23969380) exhibited substantial heterogeneity (I^2^ = 66.5% and 63%; Q-test p = 0.0297 and 0.0437, respectively), suggesting possible cohort-specific variation. In contrast, the *UST* (cg02561343) CpG showed no evidence of heterogeneity (I^2^ = 0%; Q-test p = 0.8237), indicating consistent effects across cohorts (Supplementary Table S14).

#### Model collinearity assessment

To assess potential multicollinearity among model covariates, pairwise correlations and variance inflation factors (VIFs) were calculated for the RODAM and AADM EWAS model. For RODAM, log-transformed adiponectin and BMI were moderately correlated (r = −0.38, p < 0.001), consistent with expected physiological relationships. All adjusted VIF values were <10, with most below 5 (Supplementary Table S15), indicating no problematic collinearity. In AADM, log-transformed adiponectin and BMI were similarly moderately correlated (r = −0.38, p < 0.001), and all adjusted VIF values were <10, with most below 5 (Supplementary Table S15), indicating no problematic collinearity. Overall, no evidence of problematic multicollinearity was detected in either cohort.

## Discussion

In this epigenome-wide association study of 908 West Africans from Ghana and Nigeria, we identified CpG sites associated with circulating adiponectin levels. Three CpGs, annotated to *FKBP5* (cg03546163), *UST* (cg02561343), and *ADGRD1* (cg23969380), were associated with adiponectin levels in the combined West African analysis, across T2D status. Population- and T2D-stratified analyses revealed additional distinct signals. Two differentially methylated regions were mapped to *RNF39* and *NINJ2*, and eQTM analysis linked key CpGs to expression of *PLA2G12B*, *PSMD8*, *TECR*, and *HIGD2AP1*.

The strongest and most consistent association in our study was observed for cg03546163 (*FKBP5*), which exhibited a robust inverse relationship with circulating adiponectin levels across all subgroups. *FKBP5* encodes a co-chaperone of the glucocorticoid receptor complex and is a well-established regulator of glucocorticoid sensitivity, playing a central role in the physiological stress response, metabolic regulation, and inflammation.^44^^,^^45^ Glucocorticoid signalling directly influences adipose tissue homoeostasis, including insulin sensitivity and inflammatory responses, processes closely linked to adiponectin biology.^46^ Notably, *FKBP5* has also been implicated in lipid and glucose metabolism through its downstream effects on adipocyte function.^47^ In line with this, *FKBP5* is annotated in GeneCards as being associated with lipid traits and BMI (adiposity), which may reflect shared regulatory mechanisms or confounding metabolic processes. Given its role in stress reactivity and the known epigenetic sensitivity of *FKBP5* to environmental exposures such as trauma, chronic stress, and lifestyle factors (e.g., overeating or obesity), it is also possible that methylation changes at this locus may be a consequence rather than a cause of altered adiponectin levels. We therefore cannot rule out reverse or bidirectional effects in this context. Supporting the functional relevance of this epigenetic signal, our eQTM analysis demonstrated that increased methylation at cg03546163 was associated with reduced expression of *PLA2G12B* in subcutaneous white adipose tissue, a phospholipase involved in fatty acid and glucose metabolisms.^48^ Although the mutations in *PLA2G12B* described in prior mouse models may not always result in reduced gene expression, they do impair the gene's function, particularly in terms of lipoprotein assembly and secretion. Additionally, *Pla2g12b* mutant mice exhibit profound resistance to atherosclerosis,^49^ likely due to impaired lipoprotein secretion leading to reduced plasma triglyceride levels. This supports the concept of an evolutionary trade-off where increased efficiency in triglyceride transport may enhance energy delivery under nutrient-scarce conditions, but at the cost of elevated cardiovascular risk in modern metabolic environments. Functional follow-up analyses are needed to better understand the biological mechanisms, especially given the modest effect size of the *PLA2G12B* eQTM.

Methylation at cg02561343 (*UST*) showed associations with the expression of both *PSMD8* and *TECR*, suggesting a link with protein turnover and lipid metabolism in adiponectin regulation. Our eQTM analysis revealed that higher methylation at cg02561343 was associated with reduced expression of *PSMD8*, a subunit of the proteasome complex expressed in adipose tissue.^50^ While *PSMD8* has not been directly implicated in adiponectin pathways, the proteasome plays a key role in regulating protein turnover, including that of adipokines.^51^ Specifically, proteasomes have been shown to degrade adiponectin multimers; thus, lower *PSMD8* expression could stabilise high-molecular-weight adiponectin, the most biologically active form, thereby enhancing its insulin-sensitising and anti-inflammatory effects. This stabilisation may contribute to the observed positive association between *UST* methylation and elevated circulating adiponectin levels in this study. Concurrently, cg02561343 methylation was also linked to reduced expression of *TECR*, a gene involved in the synthesis of very-long-chain fatty acids and lipid metabolism.^52^ *TECR* plays an essential role in endothelial cell function and vascular integrity, with knockout studies demonstrating impaired angiogenesis.^52^ Of particular interest is *TECR*'s association with omega-3 fatty acids, which inhibit caveolae vesicle formation, a key process in adiponectin signalling.^52^ The dual regulatory effect of *UST* methylation on both *PSMD8* and *TECR* suggests a potential coordinated epigenetic influence, which may impact the proteasomal degradation of adiponectin and its regulation of lipid metabolism pathways. Together, these findings suggest that differential cg02561343 methylation may act as an epigenetic modulator of adiponectin functions, integrating protein turnover and lipid homoeostasis to regulate metabolic and inflammatory outcomes, including improved insulin sensitivity, reduced macrophage infiltration in adipose tissue, and lower circulating levels of pro-inflammatory cytokines such as TNF-α and IL-6.

The third significant CpG site identified in our DMP meta-analysis, cg23969380 (*ADGRD1*), demonstrated a consistent positive association with adiponectin levels, driven by the Nigerian population, where it reached genome-wide significance. *ADGRD1*, encoding a G-protein-coupled receptor, has limited prior links to metabolic regulation, but emerging evidence suggests its involvement in cellular signalling and adipose tissue function.^53^^,^^54^ Methylation at cg23969380 was significantly associated with reduced expression of *HIGD2AP1* in blood. Although *HIGD2AP1* is annotated as a pseudogene and does not encode a protein, pseudogenes can exert regulatory roles through various mechanisms such as acting as competing endogenous RNAs or influencing chromatin architecture. This interpretation was informed by the known role of *HIGD2A*, a separate protein-coding gene involved in mitochondrial complex assembly and oxidative stress adaptation.^55^ However, because *HIGD2AP1* and *HIGD2A* are located on different chromosomes (2 and 5, respectively) and no direct functional relationship is established between them, we interpret the observed association with *HIGD2AP1* expression cautiously. It is possible that *HIGD2AP1* transcription reflects broader transcriptional or chromatin responses linked to stress or metabolic state, rather than direct involvement in mitochondrial function. Thus, the link between *ADGRD1* methylation and *HIGD2AP1* expression may signal a novel, yet uncharacterised, regulatory process with potential relevance to adiponectin biology. Further studies are needed to understand the functional implications of this association, particularly in the context of non-coding RNA regulation and cellular stress adaptation in metabolic disease.

Our stratified analyses by T2D status did not yield any epigenome-wide significant CpG associations; however, several of the top-ranked signals remained directionally consistent with findings from the overall analysis, suggesting shared epigenetic patterns across glycaemic strata. The lack of epigenome-wide significant findings in the T2D-stratified models is likely multifactorial. Methodologically, stratification substantially reduced the sample size within each group, limiting statistical power to detect modest effects. Furthermore, individuals with established T2D may exhibit greater metabolic and treatment-related heterogeneity (e.g. use of glucose-lowering medication, disease duration, or secondary metabolic adaptations), which could obscure methylation–adiponectin associations. Biologically, differential methylation may be more prominent in pre-disease or non-diabetic states, where adiponectin regulation reflects early metabolic shifts rather than downstream disease processes. Thus, the stronger signals observed in the non-T2D subgroup likely capture upstream regulatory mechanisms preceding overt dysglycaemia, consistent with the notion that adiponectin-related methylation marks may contribute to metabolic susceptibility rather than manifest disease.

We identified two DMRs, mapped to *RNF39* and *NINJ2*, linked to adiponectin regulation and metabolic health. Methylation status at *RNF39* has previously been implicated in adiponectin regulation, with methylation at this locus shown to be inversely associated with adiponectin levels in response to dietary interventions,^56^ highlighting its potential role in environmentally modifiable pathways linked to adiponectin biology. Meanwhile, *NINJ2*, primarily known for its role in neuroinflammation, has emerging relevance in metabolic health through its involvement in adipocyte differentiation.^57^ The differential methylation pattern observed in *NINJ2* may indicate reduced adipocyte differentiation, which could contribute to lower serum adiponectin levels. In turn, this hypoadiponectinaemia may reflect impaired adiponectin synthesis and/or secretion. These DMRs were population specific, with *RNF39* identified in Ghanaians and *NINJ2* in Nigerians, which highlights the need for inclusion of diverse populations for discovery of epigenetic regulation with relevance to biology. Beyond population-specific findings, the meta-level analysis revealed coordinated regional methylation changes implicating genes involved in transcriptional regulation, oxidative stress, and metabolic signalling. Regions annotated to *TP73/WDR8*, *EYA4*, *HOXD8*, and *GULP1* suggest links to cellular stress responses and endocrine function, while *EDNRB* and *EPB41L3* point to roles in vascular integrity and lipid metabolism. Notably, enrichment of neural and developmental genes such as *FOXG1*, *GABRA2*, and *NKX6-2* supports emerging evidence that adiponectin also modulates neurometabolic and central energy regulatory pathways. Collectively, these results indicate that adiponectin-associated methylation acts through integrated immune-metabolic and neuroendocrine networks, reflecting coordinated epigenetic regulation of inflammation, lipid handling, and cellular stress resilience.^58^, ^59^, ^60^

Beyond locus–level associations, our integrative in-silico analyses provide further insight into the potential regulatory mechanisms linking methylation to adiponectin biology. The enrichment of adiponectin-associated CpGs in enhancer and promoter-flanking chromatin regions marked by H3K27ac and H3K4me1 particularly within adipose tissue, blood, and skeletal muscle supports their localisation within transcriptionally active domains that coordinate metabolic regulation. Such enrichment patterns are consistent with adiponectin's pleiotropic roles in lipid metabolism, insulin sensitivity, and inflammatory control. Furthermore, the observation that hypermethylation of *FKBP5* and *UST* corresponded with lower gene expression, while *ADGRD1* showed similar tissue-specific regulation, suggests that these epigenetic marks may exert transcriptional effects rather than act as passive biomarkers. Collectively, these findings, together with the pathway enrichments highlighting cardiovascular and neuroendocrine processes, suggest that adiponectin-associated methylation may influence systemic energy homoeostasis through interconnected stress, inflammatory, and metabolic signalling pathways.

At the pathway level, functional enrichment analyses revealed distinct but complementary biological themes across cohorts. The cardiovascular enrichment observed in RODAM is consistent with adiponectin's established roles in myocardial energy metabolism, vascular protection, and endothelial signalling. In contrast, the strong enrichment of neurodevelopmental and synaptic pathways identified in AADM suggests a potential involvement of adiponectin in neurometabolic regulation and central signalling processes, aligning with emerging evidence linking adiponectin to brain energy homoeostasis and neuronal plasticity. These results highlight both shared and population-specific methylation signatures, providing a more nuanced picture of adiponectin's multisystem influence across cardiometabolic and neuroendocrine domains.

Our findings suggest both population (Ghanaians vs. Nigerians) and geographic (Ghanaians resident in Amsterdam vs. urban or rural Ghana) variation in the epigenetic regulation of adiponectin levels. These differences may reflect environmental exposures linked to migration, urbanisation, dietary patterns, or psychosocial stress–factors that have previously been shown in the RODAM study to influence DNA methylation profiles.^61^^,^^62^ Importantly, our analytical models accounted for potential environmental and study-level confounding by adjusting for recruitment site within the RODAM cohort and performing cohort-stratified EWAS followed by meta-analysis across studies. This approach mitigates the risk that observed differences are primarily driven by cohort-specific or migration-related factors. Nevertheless, residual confounding by unmeasured variables such as diet, socioeconomic status, or other environmental exposures cannot be entirely excluded. Furthermore, while a prior EWAS of adiponectin in European and Asian populations reported one significant CpG site annotated to the *CPT1A* gene,^22^ this locus did not reach significance in our study. The observed population differences not only reinforce the sensitivity of epigenetic marks to environmental context but also highlight the critical need for ensuring the inclusion of diverse populations from various genetic and environmental backgrounds. The discovery of both common and population-specific, epigenetic loci associated with adiponectin levels leveraging cohorts of West Africans contributes to a more comprehensive understanding of its role in metabolic regulation. Our study paves the way for future research to replicate and validate these signals and explore their translational potential in addressing the growing burden of metabolic disease across populations in sub-Saharan Africa and beyond.

### Strengths and limitations

One of the key strengths of our study is the inclusion of two large, West African cohorts, which allowed for the identification of epigenetic signals. While the RODAM-Pros subset used for epigenetic profiling was modest in size, the sample was originally powered to detect meaningful methylation differences between diabetic and non-diabetic individuals, ensuring adequate precision for the present analyses. The complementary inclusion of the larger AADM cohort and the subsequent meta-analytic approach enhanced statistical power and improved generalisability. Importantly, although the total sample size is relatively modest by EWAS standards, it is substantially larger than the only previous EWAS of adiponectin in African-ancestry individuals, which included just 243 participants of African descent, underscoring the relative strength and unique contribution of our study to the field. Nevertheless, results from smaller stratified subgroups, such as those based on T2D status, should be interpreted with caution given reduced sample size and potential sampling variability. Notably, our study identified genome-wide significant methylation loci associated with circulating adiponectin levels, demonstrating that the available sample size was sufficient to detect biologically meaningful associations. The use of harmonised epigenetic analysis pipelines, rigorous quality control procedures, and a meta-analysis approach enhanced the robustness of our results. Moreover, the functional annotation through eQTM analysis using data derived from the same populations provided valuable biological context, linking methylation changes to gene expression and relevant metabolic pathways. Our findings, particularly the identification of methylation sites annotated to *FKBP5*, *UST*, and *ADGRD1*, add insight into the molecular regulation of adiponectin and reinforce the biological plausibility of these associations through connections with inflammation, lipid metabolism, and stress signalling pathways.

Despite these strengths, several limitations should be considered. The cross-sectional nature of our study limits our ability to infer causal relationships between methylation and adiponectin levels. Although our findings are consistent with known biological processes, the directionality of the associations, whether methylation affects adiponectin or vice versa, remains unclear and may vary by CpG. Few studies suggest that most CpGs are not causal, and further research using longitudinal or intervention-based designs is needed to explore this aspect. Moreover, functional validation of the identified CpGs is currently lacking. Future experimental studies such as targeted methylation editing or adipocyte differentiation assays focussing on *FKBP5*, *UST,* and *ADGRD1* will be crucial to determine whether these epigenetic changes exert causal regulatory effects on adiponectin expression and secretion. Additionally, the use of different DNA methylation arrays between the RODAM and AADM cohorts meant that non-overlapping DNA methylation sites were not evaluated in combined meta-analyses. As this was a population-based study, DNA methylation was measured in peripheral blood, which may not fully capture tissue-specific regulatory mechanisms occurring in adipose tissue, the primary site of adiponectin synthesis. However, blood-based methylation signatures are valuable for identifying systemic epigenetic variation linked to metabolic and inflammatory pathways and have been shown to correlate with adipose methylation at several loci. Moreover, our integration of eQTM data from subcutaneous adipose tissue provides partial validation of tissue relevance, strengthening the biological interpretation of our findings. Future studies leveraging adipose or multi-tissue methylation and transcriptomic data will be essential to establish tissue-specific regulatory mechanisms and causal relationships. Although differences between the RODAM and AADM cohorts were observed, formal heterogeneity analyses indicated that only two CpGs (*FKBP5* and *ADGRD1*) showed moderate-to-high heterogeneity, while the *UST* locus was highly consistent across cohorts. However, with only two cohorts from distinct populations (RODAM Ghanaians and AADM Nigerians), we cannot distinguish whether these difference are driven by cohort-specific factors or by population-specific ancestry or environmental exposures. Additionally, while further stratification by BMI or adiponectin quartiles could potentially reveal subgroup-specific associations, these analyses would have reduced statistical power given the modest sample size. Instead, BMI was included as a covariate in all models to minimise confounding and to isolate methylation effects independent of adiposity. Finally, while the eQTM findings linking *FKBP5, UST*, and *ADGRD1* to gene expression in relevant metabolic tissues provide in-silico functional support, experimental validation is required to confirm causality. Future studies employing CRISPR-based methylation editing, adipocyte differentiation models, or reporter assays are warranted to determine whether methylation at these loci directly modulates adiponectin-related gene expression and metabolic function. Lastly, total adiponectin was measured, which does not allow us to distinguish the biologically active high-molecular-weight (HMW) form from other isoforms of adiponectin.

### Conclusions

We identified epigenome-wide associations for circulating adiponectin leveraging two unique cohorts of West African populations, particularly at *FKBP5*, *UST*, and *ADGRD1*. These findings expand current knowledge of adiponectin regulation and highlight the importance of studying diverse populations for discovery of DNA methylation markers. Further research is needed to confirm these findings in other populations, and to explore the functional consequences of these methylation changes using longitudinal and experimental approaches.

## Contributors

MMM, JW, and KACM conceived and designed the study. MMM and JW analysed the data under the supervision of KACM. MMM wrote the manuscript together with KACM. MMM and KACM accessed and verified the underlying data. All authors contributed to data interpretation, critically revised the manuscript, and read and approved the final version of the manuscript.

## Data sharing statement

The data supporting the findings of this study are available in part through repositories and in part upon reasonable request as part of a collaboration.

Summary statistics from the epigenome-wide association meta-analysis of circulating adiponectin in West Africans are provided as supplementary files with this manuscript and are also deposited in an external public repository (Figshare: https://doi.org/10.6084/m9.figshare.31229737).

Summary statistics from the eQTM analyses are available through the database of Genotypes and Phenotypes (dbGaP; accession number phs001844.v2.p1). Analysis code used for data processing and statistical analyses is available via a public GitHub repository (https://github.com/NHGRI/CRGGH/tree/Mungamba_DNAm_Adiponectin_RODAM_AADM), with an archived version to be made available at the time of publication.

Due to privacy and ethical considerations, individual-level data cannot be made publicly available. RNA-sequencing data from the AADM study used to generate the eQTMs will be available via controlled access in dbGaP (accession number phs001844.v2.p1), in accordance with participant consent and ethical approvals. Requests for access to other individual-level data should be directed to the Principal Investigators of the RODAM study (c.o.agyemang@amsterdamumc.nl) or the AADM study (rotimic@mail.nih.gov) and will be reviewed by the scientific committees of the RODAM and AADM studies. The data are securely stored and can be accessed in accordance with the ethical guidelines set by the RODAM and AADM studies, as well as the participating institutions, upon reasonable request.

## Declaration of interests

The authors declare no competing interests.

## References

[1] Tian L., Luo N., Klein R.L., Chung B.H., Garvey W.T., Fu Y. (2009). Adiponectin reduces lipid accumulation in macrophage foam cells. Atherosclerosis.

[2] Furukawa K., Hori M., Ouchi N. (2004). Adiponectin down-regulates acyl-coenzyme A: cholesterol acyltransferase-1 in cultured human monocyte-derived macrophages. Biochem Biophys Res Commun.

[3] Li S., Shin H.J., Ding E.L., van Dam R.M. (2009). Adiponectin levels and risk of type 2 diabetes: a systematic review and meta-analysis. JAMA.

[4] Hao G., Li W., Guo R. (2013). Serum total adiponectin level and the risk of cardiovascular disease in general population: a meta-analysis of 17 prospective studies. Atherosclerosis.

[5] Yuyun M.F., Sliwa K., Kengne A.P., Mocumbi A.O., Bukhman G. (2020). Cardiovascular diseases in Sub-Saharan Africa compared to high-income countries: an epidemiological perspective. Glob Heart.

[6] Cappuccio F.P., Miller M.A. (2016). Cardiovascular disease and hypertension in Sub-Saharan Africa: burden, risk and interventions. Intern Emerg Med.

[7] Mbanya J.C., Assah F.K., Saji J., Atanga E.N. (2014). Obesity and type 2 diabetes in Sub-Sahara Africa. Curr Diab Rep.

[8] Vasseur F., Helbecque N., Dina C. (2002). Single-nucleotide polymorphism haplotypes in the both proximal promoter and exon 3 of the APM1 gene modulate adipocyte-secreted adiponectin hormone levels and contribute to the genetic risk for type 2 diabetes in French Caucasians. Hum Mol Genet.

[9] Cesari M., Narkiewicz K., De Toni R., Aldighieri E., Williams C.J., Rossi G.P. (2007). Heritability of plasma adiponectin levels and body mass index in twins. J Clin Endocrinol Metab.

[10] Liu P.-H., Jiang Y.-D., Chen W.J. (2008). Genetic and environmental influences on adiponectin, leptin, and BMI among adolescents in Taiwan: a multivariate twin/sibling analysis. Twin Res Hum Genet.

[11] Spracklen C.N., Iyengar A.K., Vadlamudi S. (2020). Adiponectin GWAS loci harboring extensive allelic heterogeneity exhibit distinct molecular consequences. PLoS Genet.

[12] Erlandsson M.C., Doria Medina R., Töyrä Silfverswärd S., Bokarewa M.I. (2016). Smoking functions as a negative regulator of IGF1 and impairs adipokine network in patients with rheumatoid arthritis. Mediators Inflamm.

[13] Kuusisto S.M., Peltola T., Laitinen M. (2012). The interplay between lipoprotein phenotypes, adiponectin, and alcohol consumption. Ann Med.

[14] Nishise Y., Saito T., Makino N. (2010). Relationship between alcohol consumption and serum adiponectin levels: the Takahata study—a cross-sectional study of a healthy Japanese population. J Clin Endocrinol Metab.

[15] Sirico F., Bianco A., D'Alicandro G. (2018). Effects of physical exercise on adiponectin, leptin, and inflammatory markers in childhood obesity: systematic review and meta-analysis. Child Obes.

[16] Khalil W.J., Akeblersane M., Khan A.S., Moin A.S.M., Butler A.E. (2023). Environmental pollution and the risk of developing metabolic disorders: obesity and diabetes. Int J Mol Sci.

[17] Cartwright A. (2015).

[18] Mattei A.L., Bailly N., Meissner A. (2022). DNA methylation: a historical perspective. Trends Genet.

[19] Kim A.Y., Park Y.J., Pan X. (2015). Obesity-induced DNA hypermethylation of the adiponectin gene mediates insulin resistance. Nat Commun.

[20] Parker-Duffen J.L., Walsh K. (2014). Cardiometabolic effects of adiponectin. Best Pract Res Clin Endocrinol Metab.

[21] Iwashima Y., Katsuya T., Ishikawa K. (2004). Hypoadiponectinemia is an independent risk factor for hypertension. Hypertension.

[22] Aslibekyan S., Do A., Xu H. (2017). CPT1A methylation is associated with plasma adiponectin. Nutr Metab Cardiovasc Dis.

[23] Lai C.-Q., Parnell L.D., Smith C.E. (2020). Carbohydrate and fat intake associated with risk of metabolic diseases through epigenetics of CPT1A. Am J Clin Nutr.

[24] Meilleur K.G., Doumatey A., Huang H. (2010). Circulating adiponectin is associated with obesity and serum lipids in West Africans. J Clin Endocrinol Metab.

[25] Schuster D.P., Gaillard T., Osei K. (2007). The cardiometabolic syndrome in persons of the African Diaspora: challenges and opportunities. J Cardiometab Syndr.

[26] Lomakova Y.D., Chen X., Stein T.P., Steer R.A. (2022). Decreased adiponectin levels in early pregnancy are associated with high risk of prematurity for African American women. J Clin Med.

[27] Agyemang C., van der Linden E.L., Antwi-Berko D. (2022). Cohort profile: Research on Obesity and Diabetes among African Migrants in Europe and Africa Prospective (RODAM-Pros) cohort study. BMJ Open.

[28] Agyemang C., Beune E., Meeks K. (2014). Rationale and cross-sectional study design of the research on obesity and type 2 diabetes among African migrants: the RODAM study. BMJ Open.

[29] Swarbrick M.M., Havel P.J. (2008). Physiological, pharmacological, and nutritional regulation of circulating adiponectin concentrations in humans. Metab Syndr Relat Disord.

[30] Rotimi C.N., Dunston G.M., Berg K. (2001). In search of susceptibility genes for type 2 diabetes in West Africa: the design and results of the first phase of the AADM study. Ann Epidemiol.

[31] Doumatey A.P., Feron H., Ekoru K., Zhou J., Adeyemo A., Rotimi C.N. (2021). Serum fructosamine and glycemic status in the presence of the sickle cell mutation. Diabetes Res Clin Pract.

[32] Du P., Zhang X., Huang C.-C. (2010). Comparison of beta-value and M-value methods for quantifying methylation levels by microarray analysis. BMC Bioinformatics.

[33] Houseman E.A., Accomando W.P., Koestler D.C. (2012). DNA methylation arrays as surrogate measures of cell mixture distribution. BMC Bioinformatics.

[34] Li B., Dewey C.N. (2011). RSEM: accurate transcript quantification from RNA-Seq data with or without a reference genome. BMC Bioinformatics.

[35] Langmead B., Wilks C., Antonescu V., Charles R. (2019). Scaling read aligners to hundreds of threads on general-purpose processors. Bioinformatics.

[36] Meeks K.A., Henneman P., Venema A. (2019). Epigenome-wide association study in whole blood on type 2 diabetes among Sub-Saharan African individuals: findings from the RODAM study. Int J Epidemiol.

[37] Willer C.J., Li Y., Abecasis G.R. (2010). METAL: fast and efficient meta-analysis of genomewide association scans. Bioinformatics.

[38] Jaffe A.E., Murakami P., Lee H. (2012). Bump hunting to identify differentially methylated regions in epigenetic epidemiology studies. Int J Epidemiol.

[39] Suderman M., Staley J.R., French R., Arathimos R., Simpkin A., Tilling K. (2018). Dmrff: identifying differentially methylated regions efficiently with power and control. BioRxiv.

[40] Komaki S., Shiwa Y., Furukawa R. (2018). iMETHYL: an integrative database of human DNA methylation, gene expression, and genomic variation. Hum Genome Var.

[41] Stelzer G., Rosen N., Plaschkes I. (2016). The GeneCards suite: from gene data mining to disease genome sequence analyses. Curr Protoc Bioinformatics.

[42] Sayers E.W., Beck J., Bolton E.E. (2025). Database resources of the National Center for Biotechnology Information in 2025. Nucleic Acids Res.

[43] Li M., Zou D., Li Z. (2019). EWAS Atlas: a curated knowledgebase of epigenome-wide association studies. Nucleic Acids Res.

[44] Binder E.B. (2009). The role of FKBP5, a co-chaperone of the glucocorticoid receptor in the pathogenesis and therapy of affective and anxiety disorders. Psychoneuroendocrinology.

[45] Zannas A.S., Jia M., Hafner K. (2019). Epigenetic upregulation of FKBP5 by aging and stress contributes to NF-κB–driven inflammation and cardiovascular risk. Proc Natl Acad Sci U S A.

[46] Lee R.A., Harris C.A., Wang J.-C. (2018). Glucocorticoid receptor and adipocyte biology. Nucl Receptor Res.

[47] Sidibeh C.O., Pereira M.J., Abalo X.M. (2018). FKBP5 expression in human adipose tissue: potential role in glucose and lipid metabolism, adipogenesis and type 2 diabetes. Endocrine.

[48] Wu M., Wang Q., Li H. (2024). PLA2G12A protects against diet-induced obesity and insulin resistance by enhancing energy expenditure and clearance of circulating triglycerides. FASEB J.

[49] Thierer J.H., Foresti O., Yadav P.K. (2024). Pla2g12b drives expansion of triglyceride-rich lipoproteins. Nat Commun.

[50] Zhu Y., Li N., Huang M. (2021). Adipose tissue hyaluronan production improves systemic glucose homeostasis and primes adipocytes for CL 316,243-stimulated lipolysis. Nat Commun.

[51] Willemsen N., Arigoni I., Studencka-Turski M., Krüger E., Bartelt A. (2022). Proteasome dysfunction disrupts adipogenesis and induces inflammation via ATF3. Mol Metab.

[52] Wang J., Xu J., Zang G. (2022). Trans-2-enoyl-CoA reductase Tecr-driven lipid metabolism in endothelial cells protects against transcytosis to maintain blood-brain barrier homeostasis. Research (Wash D C).

[53] Al Mahri S., Okla M., Rashid M. (2023). Profiling of G-protein coupled receptors in adipose tissue and differentiating adipocytes offers a translational resource for obesity/metabolic research. Cells.

[54] Im H., Park J.-H., Im S., Han J., Kim K., Lee Y.-H. (2021). Regulatory roles of G-protein coupled receptors in adipose tissue metabolism and their therapeutic potential. Arch Pharm Res.

[55] Salazar C., Barros M., Elorza A.A., Ruiz L.M. (2021). Dynamic distribution of HIG2A between the mitochondria and the nucleus in response to hypoxia and oxidative stress. Int J Mol Sci.

[56] Ramos-Lopez O. (2023). Epigenetic biomarkers of metabolic responses to lifestyle interventions. Nutrients.

[57] Peng H., Yu Y., Wang P. (2023). NINJ2 deficiency inhibits preadipocyte differentiation and promotes insulin resistance through regulating insulin signaling. Obesity.

[58] Sinke L., Delerue T., Wilson R. (2025). DNA methylation of genes involved in lipid metabolism drives adiponectin levels and metabolic disease. Diabetologia.

[59] Ma X., Kang S. (2019). Functional implications of DNA methylation in adipose biology. Diabetes.

[60] McAllan L., Baranasic D., Villicaña S. (2023). Integrative genomic analyses in adipocytes implicate DNA methylation in human obesity and diabetes. Nat Commun.

[61] Chilunga F.P., Henneman P., Venema A. (2021). DNA methylation as the link between migration and the major noncommunicable diseases: the RODAM study. Epigenomics.

[62] van der Laan L.C., Meeks K.A., Chilunga F.P. (2020). Epigenome-wide association study for perceived discrimination among Sub-Saharan African migrants in Europe-the RODAM study. Sci Rep.

