# Supplementary material for: Epigenome-wide analysis in West Africans identifies DNA methylation markers for circulating adiponectin
Source: eBioMedicine. 2026 Mar 6;126:106192. doi: 10.1016/j.ebiom.2026.106192 (PMC12993010; doi:10.1016/j.ebiom.2026.106192)
Supplement: Supplementary File [file mmc1.docx]

**Supplementary Material**

**Supplement to:**

Mungamba MM, Wijburg J, van der Linden et al. *Epigenome-wide analysis in West Africans identifies DNA methylation markers for circulating adiponectin.*

**Contents:**

**Supplementary Figure S1:** QQ-plots of genome-wide p-values for meta-analysis of Nigerian and Ghanaian participants combined, and separately A) Combined Nigerian and Ghanaian T2D cases and controls (n = 908), B) Nigerian T2D cases and controls (AADM; n = 593), and C) Ghanaian T2D cases and controls (RODAM-Pros; n = 315).

**Supplementary Figure S2:** QQ plots of genome-wide p-values from the meta-analysis of Nigerian (AADM; n = 593) and Ghanaian participants (RODAM-Pros; n = 315) with T2D cases and controls analysed separately.

**Supplementary Figure S3:** QQ plots of genome-wide p-values for Nigerians (AADM; n = 593) and Ghanaians (RODAM-Pros; n = 315) analysed separately, stratified by T2D status (cases and controls).

**Supplementary Figure S4:** Manhattan plot for meta-analysis of A) Ghanaian and Nigerian participants with T2D (n = 389; AADM: 277, RODAM-Pros: 112), and B) Ghanaian and Nigerian non-T2D participants (n = 519; AADM: 316, RODAM-Pros: 203).

**Supplementary Figure S5:** Manhattan plot of epigenome-wide association analysis in Nigerian participants (AADM; n = 593; 277 T2D cases and 316 non-T2D controls), comparing those with and without T2D.

**Supplementary Figure S6:** Manhattan plot of epigenome-wide association analysis in Ghanaian participants (RODAM-Pros; n = 315; 112 T2D cases and 203 non-T2D controls), comparing those with and without T2D.

**Supplementary Results S1:** Adiponectin-associated DMPs stratified by T2D status

**Supplementary Table S1**: Full list of significant differentially methylated regions (DMRs) identified using DMRff in the combined meta-analysis across AADM and RODAM cohorts (FDR < 0.05).

**Supplementary Table S2:** Gene Ontology (GO) enrichment results for the top 5,000 CpGs in the RODAM cohort (*missMethyl* analysis).

**Supplementary Table S3:** GO enrichment results for the top 1,000 CpGs in the RODAM cohort (*missMethyl* analysis).

**Supplementary Table S4:** Kyoto Encyclopedia of Genes and Genomes (KEGG) pathway enrichment results for the top 5,000 CpGs in the RODAM cohort (*missMethyl* analysis).

**Supplementary Table S5:** KEGG pathway enrichment results for the top 1,000 CpGs in the RODAM cohort (*missMethyl* analysis).

**Supplementary Table S6:** GO enrichment results for the top 1,000 CpGs in the AADM cohort (*missMethyl* analysis).

**Supplementary Table S7:** GO enrichment results for the top 5,000 CpGs in the AADM cohort (*missMethyl* analysis).

**Supplementary Table S8:** KEGG pathway enrichment results for the top 5,000 CpGs in the AADM cohort (*missMethyl* analysis).

**Supplementary Table S9:** KEGG pathway enrichment results for the top 1,000 CpGs in the AADM cohort (*missMethyl* analysis).

**Supplementary Table S10:** Gene Ontology enrichment results using *clusterProfiler* for the AADM cohort.

**Supplementary Table S11:** KEGG pathway enrichment results using *clusterProfiler* for the AADM cohort.

**Supplementary Table S12:** Gene Ontology enrichment results using *clusterProfiler* for the RODAM cohort.

**Supplementary Table S13:** KEGG pathway enrichment results using *clusterProfiler* for the RODAM cohort.

**Supplementary Table S14:** Assessment of cohort-level heterogeneity in EWAS meta-analysis results.

**Supplementary Table S15:** Collinearity diagnostics and model stability analyses for covariate-adjusted EWAS models.

**Supplementary Table S16:** eFORGE enrichment results for CpGs with FDR < 0.4 using Consolidated Roadmap Epigenomics – DNase I Hypersensitivity (DHS) dataset.

**Supplementary Table S17:** eFORGE enrichment results using Consolidated Roadmap Epigenomics – Chromatin (15-state model) dataset.

**Supplementary Table S18:** eFORGE enrichment results using Consolidated Roadmap Epigenomics – All H3 histone modification marks dataset.

**Supplementary Table S19:** eFORGE enrichment results using ENCODE – DNase I Hypersensitivity (DHS) dataset.

**Notes:**

All **Supplementary Tables (S1–S19)** are provided in a single separate Excel file for ease of navigation.

All pathway enrichment analyses were performed using the *missMethyl* and *clusterProfiler* R packages. Chromatin state and regulatory feature enrichment analyses were performed using the eFORGE v2.0 platform (https://eforge.altiusinstitute.org) with permutation-based FDR correction.

**
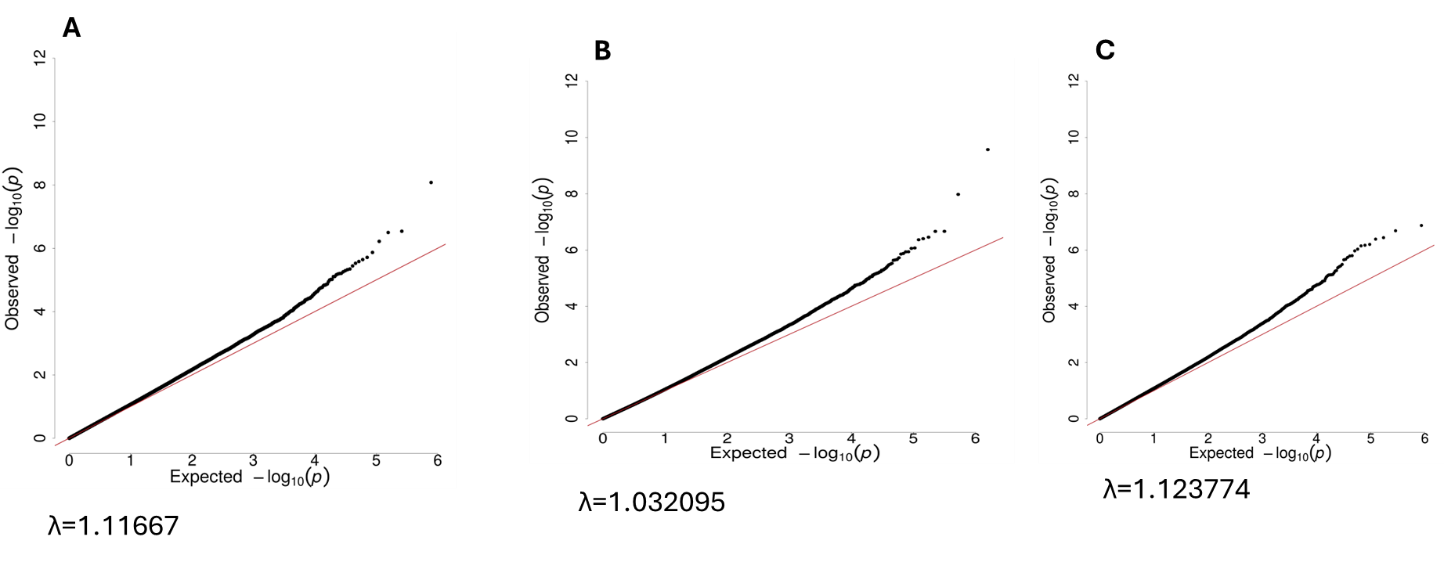
**

**Supplementary Figure S1:** QQ-plots of genome-wide p-values for meta-analysis of Nigerian and Ghanaian participants combined, and separately A) Combined Nigerian and Ghanaian T2D cases and controls (n = 908), B) Nigerian T2D cases and controls (AADM; n = 593), and C) Ghanaian T2D cases and controls (RODAM-Pros; n = 315). P-values are derived from epigenome-wide association models.

*
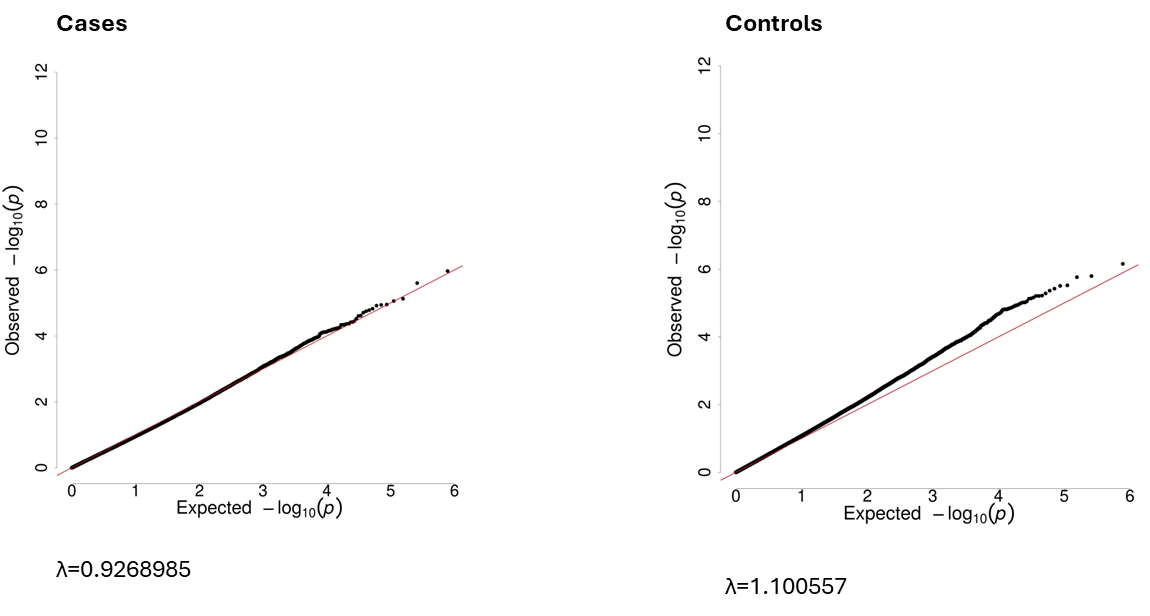
*

**Supplementary Figure S2:** QQ plots of genome-wide p-values from the meta-analysis of Nigerian (AADM; n = 593) and Ghanaian participants (RODAM-Pros; n = 315) with T2D cases and controls analysed separately.

*
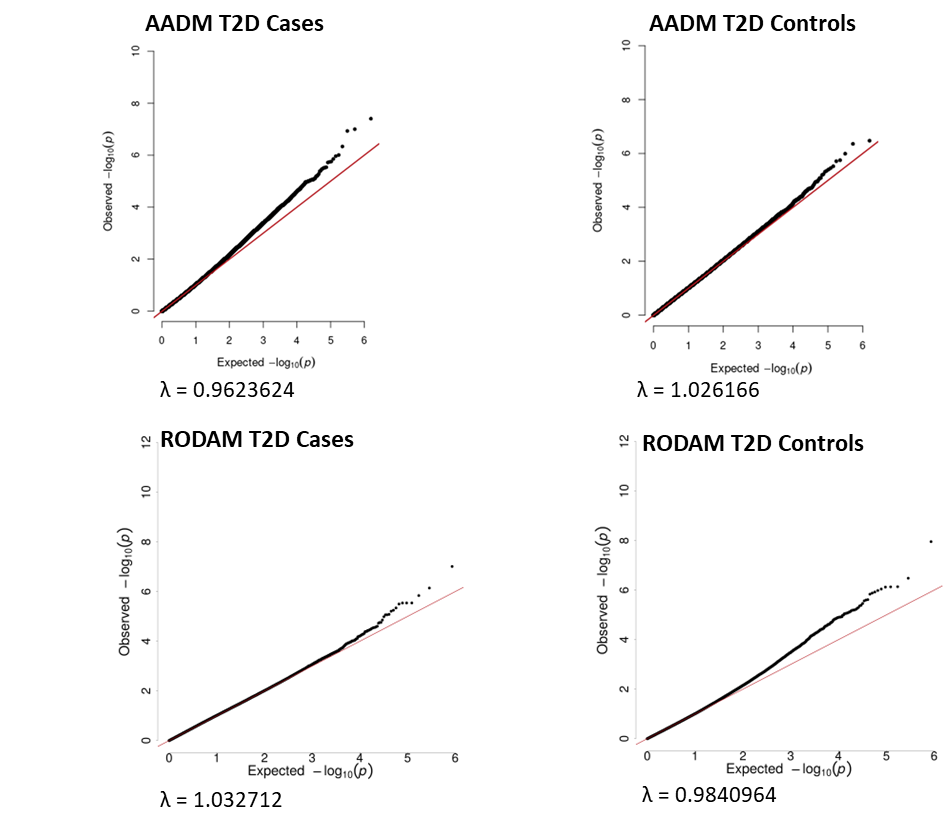
*

**Supplementary Figure S3:** QQ plots of genome-wide p-values for Nigerians (AADM; n = 593) and Ghanaians (RODAM-Pros; n = 315) analysed separately, stratified by T2D status (cases and controls).


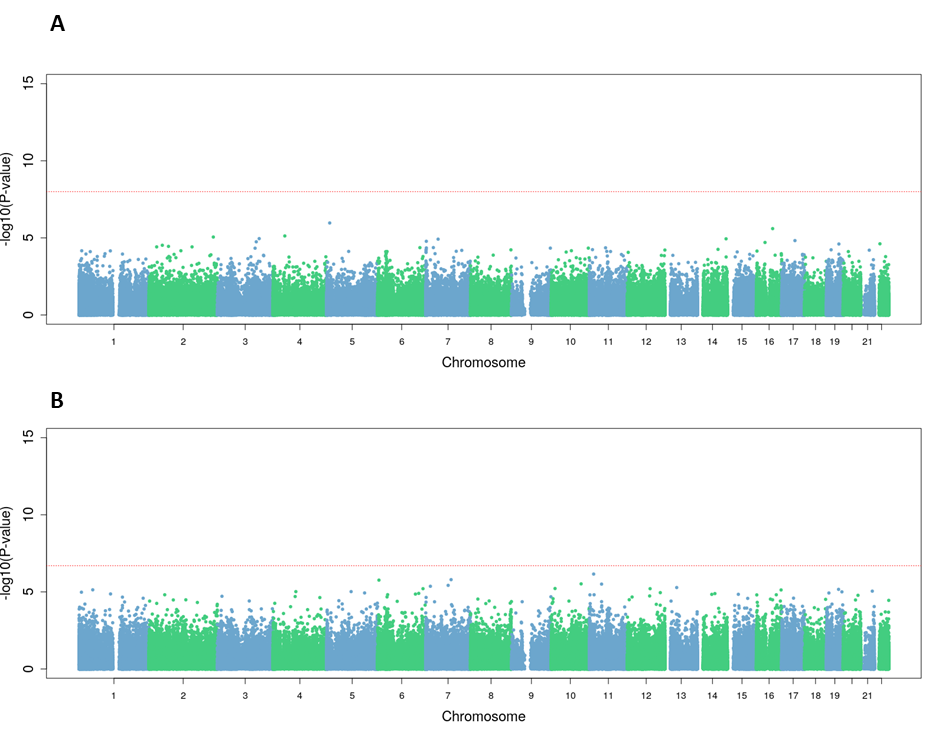


**Supplementary Figure S4:** Manhattan plot for meta-analysis of A) Ghanaian and Nigerian participants with T2D (n = 389; AADM: 277, RODAM-Pros: 112), and B) Ghanaian and Nigerian non-T2D participants (n = 519; AADM: 316, RODAM-Pros: 203). The red horizontal line indicates FDR < 0.05.


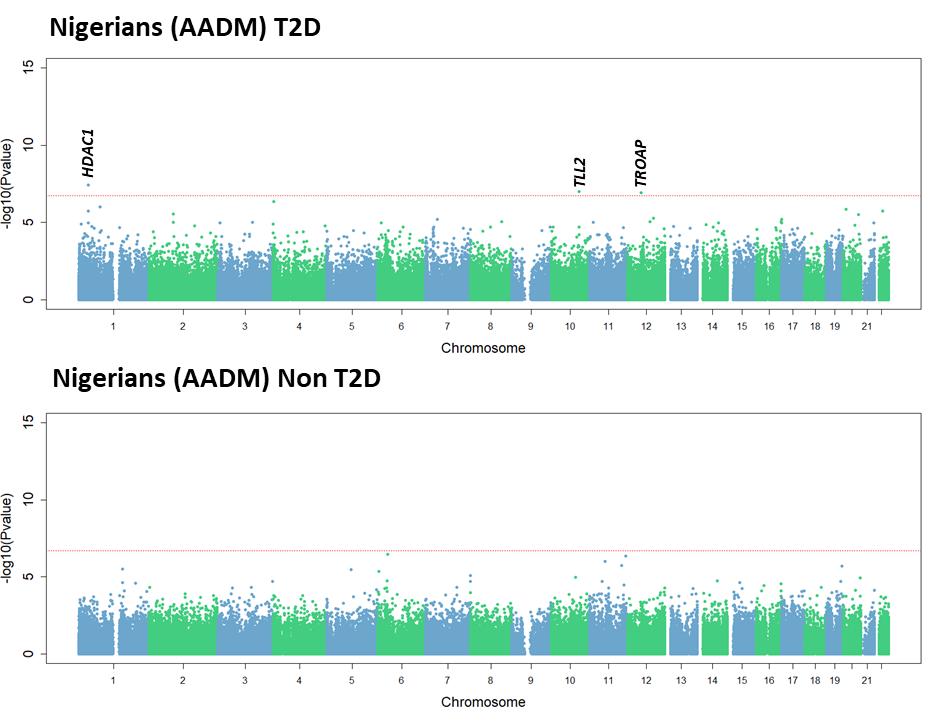


**Supplementary Figure S5:** Manhattan plot of epigenome-wide association analysis in Nigerian participants (AADM; n = 593; 277 T2D cases and 316 non-T2D controls), comparing those with and without T2D. The red horizontal line indicates FDR < 0.05.


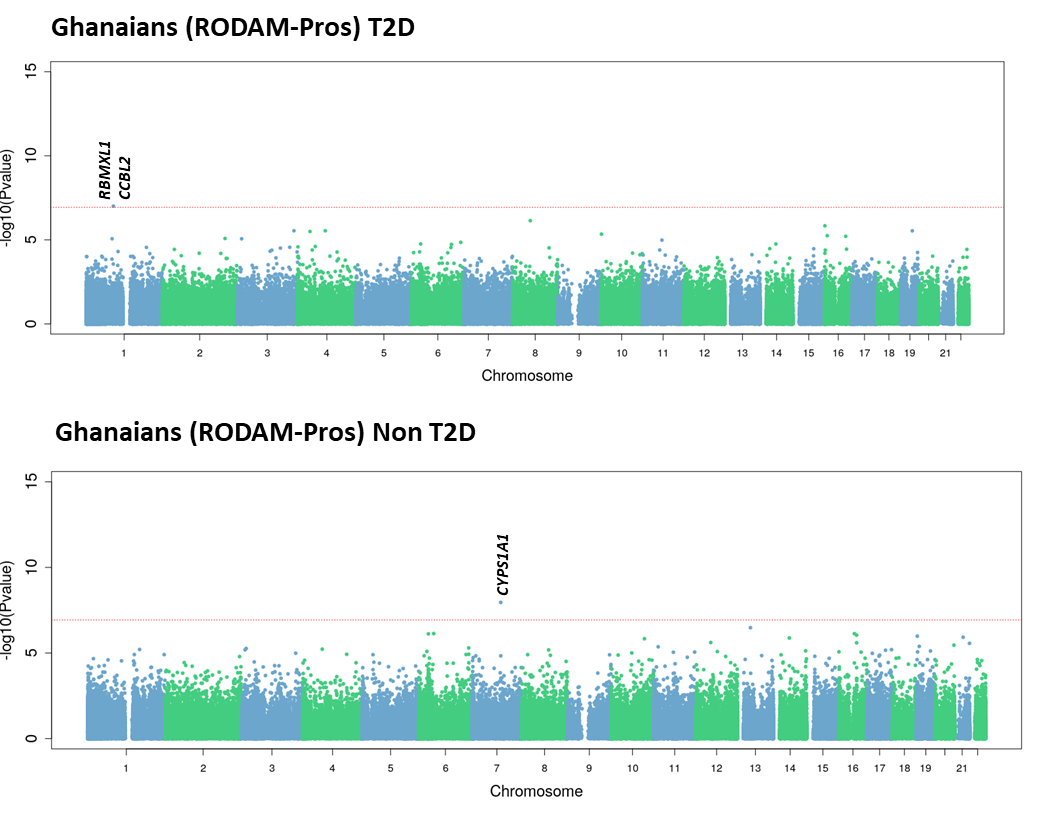


**Supplementary Figure S6:** Manhattan plot of epigenome-wide association analysis in Ghanaian participants (RODAM-Pros; n = 315; 112 T2D cases and 203 non-T2D controls), comparing those with and without T2D. The red horizontal line indicates FDR < 0.05.

**Supplementary Results S1: Adiponectin-associated DMPs stratified by T2D status**

When stratified by T2D status and cohort (Ghanaians versus Nigerians), a limited number of significant DMPs were identified; however, none overlapped with those detected in the primary meta-analyses.

In Nigerian participants with T2D, three DMPs reached epigenome-wide significance: cg10549018 (chromosome 10), cg15713729 (chromosome 1), and cg27419217 (chromosome 12), with methylation levels increasing by approximately 0.2-1.3% per unit increase in log-transformed adiponectin levels. No significant DMPs were identified among Nigerians without T2D (Supplementary Figure S5).

Among Ghanaian participants with T2D, one significant DMP was identified (cg11493241, chromosome 1), showing an approximately 2% decrease in methylation per unit increase in adiponectin. Among Ghanaians without T2D, one DMP reached significance (cg22117172, chromosome 7), with an approximately 3.5% decrease in methylation (Supplementary Figure S6).
